# Gene Therapy Targeting *Pkp2* Deficiency Attenuates Cardiac Fibrosis: Insights From Single‐Cell Transcriptomics in *Pkp2*‐Knockout Rats

**DOI:** 10.1002/mco2.70392

**Published:** 2025-09-18

**Authors:** Xinyue Ding, Hui Zhang, Xuan Zhao, Nengpin Yin, Shuo Han, Xiao Jin, Tingting Li, Lina Xing, Zhen Qi, Yanan Zhu, Xin Wang, Zongjun Liu

**Affiliations:** ^1^ Institute of Cardiovascular Translational Medicine Putuo Hospital, Shanghai University of Traditional Chinese Medicine Shanghai China; ^2^ Department of GCP Office Shanghai Ninth People's Hospital Shanghai Jiao Tong University School of Medicine Shanghai China; ^3^ Neocellmed Co. Ltd. Shanghai China; ^4^ Shanghai Key Laboratory of Regulatory Biology School of Life Sciences East China Normal University Shanghai China

**Keywords:** heart failure, myocardial fibrosis, PKP2, *Ptprc*

## Abstract

Heart failure (HF), characterized by maladaptive cardiac fibrosis and progressive functional deterioration, remains a therapeutic challenge. In this study, we established a cardiac organoid HF model derived from human‐induced pluripotent stem cells (hiPSCs) and observed a significant downregulation of the desmosomal protein plakophilin‐2 (PKP2) in this model. Reduced PKP2 expression was detected in both HF rat and mouse. Subsequent in vivo studies on *Pkp2‐*knockout (*Pkp2*‐KO) rats demonstrated that adeno‐associated virus serotype 9 (AAV9)‐mediated restoration of PKP2 not only restored cardiac PKP2 expression but also attenuated the progression of fibrosis. Administration of AAV9‐PKP2 could also inhibit myocardial fibrosis and slow down disease progression in HF mouse. Single‐cell RNA sequencing analysis in rats revealed enriched pathological profibrotic cardiac fibroblasts (CFs) in PKP2‐deficient myocardium. Mechanistically, AAV9‐PKP2 administration induced the phenotypic conversion of activated CFs into quiescent antifibrotic states. Integrated bioinformatics identified that protein tyrosine phosphatase receptor type C (*Ptprc*) was a pivotal regulator orchestrating this cellular reprogramming. Our findings thus unveil PKP2 as a master regulator of fibroblast activation and propose AAV9‐PKP2 gene therapy as a promising novel therapeutic strategy targeting pathological fibrosis in HF.

## Graphical abstract

PKP2 acts as a master switch for the progression of fibrosis in heart failure.

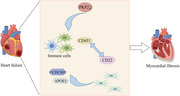

## 1 Introduction

Heart failure (HF), a terminal manifestation of diverse cardiovascular pathologies, manifests as a clinical syndrome characterized by structural cardiac remodeling and functional impairment, and myocardial fibrosis serves as a hallmark of disease progression [1, 2]. Despite etiological heterogeneity of HF, which spans ischemic injury, pressure overload, and genetic cardiomyopathies, they share common pathophysiological pathways involving myocyte apoptosis, fibrotic replacement, and electromechanical desynchrony [3]. Contemporary management strategies, including neurohormonal blockade (β‐blockers, angiotensin‐converting enzyme inhibitor [ACEI]/angiotensin II receptor blocker [ARB], angiotensin receptor‐neprilysin inhibitors [ARNIs], mineralocorticoid receptor antagonists), diuretics, and device‐based therapies, provide symptomatic relief but demonstrate limited efficacy in halting fibrotic remodeling [4, 5]. Mechanistically targeted antifibrotic interventions might be promising strategies to further improve the outcome of HF patients.

Desmosomes, specialized intercellular junctions maintaining cardiomyocyte adhesion integrity, orchestrate mechanical coupling through dynamic interactions between core structural proteins (plakophilins, desmoplakin, plakoglobin) and cytoskeletal networks [6, 7]. These junctions are indispensable for preserving myocardial syncytium integrity during the cyclic mechanical stress of cardiac contraction, and their dysfunction can precipitate structural disarray and electrical instability [8]. Although there is no direct causal relationship between HF and desmosomal structures, abnormalities in desmosomal architecture may contribute to the pathogenesis of HF in certain cardiomyopathies. Specifically, mutations or dysregulation of desmosomal proteins may disrupt intercellular connections between cardiomyocytes, compromising normal myocardial function and structural integrity [9]. For instance, in arrhythmogenic right ventricular cardiomyopathy (ARVC), genetic mutations in desmosomal components such as plakophilin‐2 (PKP2) have been identified as primary pathogenic factors [10]. These genetic alterations can induce defective intercellular adhesion and myocardial matrix remodeling, rendering cardiomyocytes more susceptible to mechanical stress‐induced dissociation and apoptosis [11]. The resultant cardiomyocyte loss and subsequent fibrofatty replacement may progressively impair cardiac contractility, ultimately culminating in HF [12]. It is observed that abnormal desmosomes play a pivotal role in the progression of HF by mediating defects in cell adhesion and promoting myocardial remodeling.

PKP2, a cardinal constituent of cardiac desmosomes, is a critical molecular scaffold that orchestrates intercellular adhesion while maintaining electromechanical coupling in cardiomyocytes [13, 14]. Beyond its structural role, PKP2 exerts pleiotropic effects on cardiac biology through modulation of the Wnt/β‐catenin signaling axis, thereby regulating cardiomyocyte proliferation, differentiation, and survival during developmental and pathological states [15, 16]. In patients with ARVC, the absence of PKP2 not only predisposes to arrhythmias but also contributes to extensive atrial fibrosis [17]. Notably, the role of PKP2 mutations in ARVC pathogenesis are well‐established, but their broader role in HF‐associated fibrosis remains underexplored. Current research indicated that PKP2 deficiency in cardiomyocytes could enhance transforming growth factor β1 (TGF‐β1) and p38 mitogen‐activated protein kinase (MAPK) signaling, promoting pro‐fibrotic gene expression [18], but their mechanistical role in fibrosis in the setting of HF has not been systemically explored. Current therapeutic paradigms lack agents capable of modulating fibroblast activation states or reversing established fibrosis, highlighting a critical translational gap in HF management. Clarifying whether PKP2 deficiency directly influences fibroblast heterogeneity or paracrine signaling between cardiomyocytes and fibroblasts could identify novel therapeutic nodes for interrupting fibrotic remodeling.

In this study, we observed a significant reduction in PKP2 protein expression in hypoxia‐injured cardiac organoid models. Notably, PKP2 replenishment therapy markedly improved cardiac function and attenuated fibrotic remodeling in post‐myocardial infarction HF mouse models, thereby providing critical evidence for the therapeutic potential of PKP2 in cardiac disorders. Prior investigations demonstrated that adeno‐associated virus serotype 9 (AAV9)‐PKP2 supplementation in c.2013delC/WT hiPSC‐derived cardiomyocytes not only restored PKP2 levels but also normalized the expression of other junctional proteins [19]. Furthermore, a single‐dose AAV9‐PKP2 gene delivery was shown to prevent disease onset when administered pre‐symptomatically and mitigate disease progression in established cardiomyopathy [20]. However, the specific impact of PKP2 deficiency on cardiac fibroblast (CFs) subpopulations remains poorly characterized. In this study, we performed single‐cell sequencing (scRNA‐seq [scRNA‐seq]) of heart tissue from wild‐type (WT) rats, *Pkp2*‐knockout (*Pkp2*‐KO) rats, and AAV9‐PKP2‐treated rats to systematically characterize the effects on CF heterogeneity following *Pkp2* knockout and reconditioning therapy.

## 2 Results

### 2.1 The Expression of PKP2 Protein Was Downregulated in Various HF Models

**FIGURE 1:**
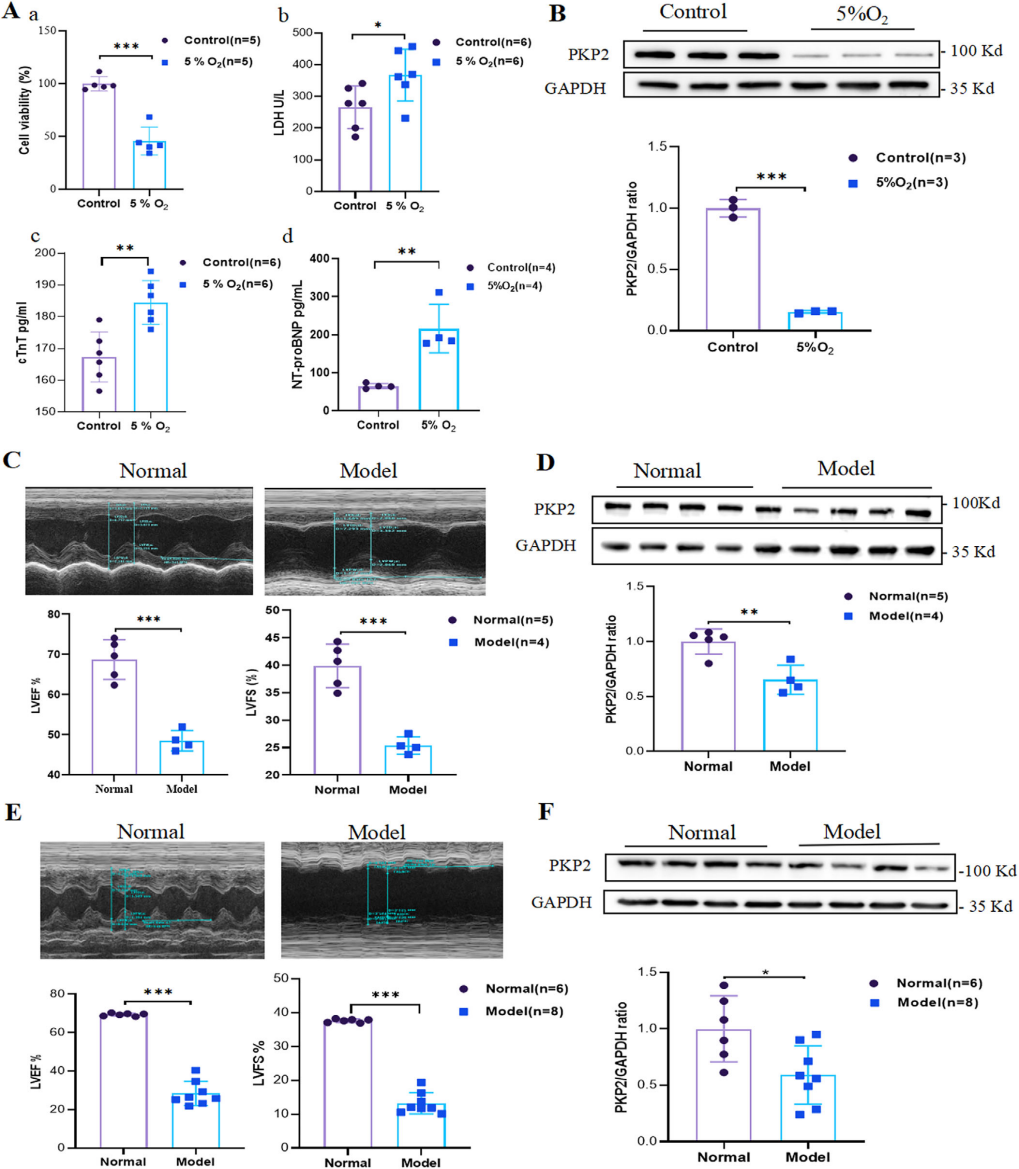
Expression of PKP2 protein in different models of heart failure (HF). (A) Detection of organoid HF modeling‐related indicators (cell viability, LDH, cTnT and NT‐proBNP) (a:n = 5, b:n = 6, c:n = 6, d:n = 4). (B) Expression of PKP2 protein in normal and hypoxia‐damaged organoids (*n* = 3). (C) Echocardiographic detection of HF‐related indicators (LVES, LVFS) in HF rats (*n* = 5:4). (D) Expression of PKP2 protein in normal and HF rats cardiac tissues (*n* = 5:4). (E) Echocardiographic detection of HF‐related indicators (LVES, LVFS) in HF mice (*n* = 6:8). (F) Expression of PKP2 protein in normal and HF mice cardiac tissues (*n* = 6:8). All data are presented as mean ± SEM. *p < 0.05, **p < 0.01 and ***p < 0.001.

In this study, we established a cardiac organoid model using hiPSCs and performed immunofluorescence validation of characteristic cardiac markers (Figure S1B). Subsequently, we established a 24‐h hypoxia‐induced cardiac organoid model of HF in vitro, with validation conducted through morphological assessment (Figure S1A) and analysis of HF markers including cell viability, lactate dehydrogenase (LDH), cardiac troponin T (cTnT), and N‐terminal pro‐B‐type Natriuretic peptide (NT‐proBNP) (Figure 1A). Western blot (WB) analysis of the HF organoid model revealed a significant reduction in PKP2 protein levels in the organoid tissues (Figure 1B), suggesting that PKP2 may play a critical role in HF models. To further investigate this, we constructed post‐myocardial infarction HF models in both rats and mice. One month after myocardial infarction induction, echocardiography was performed, and a left ventricular ejection fraction (LVEF) of less than 50% was used as the criterion for successful modeling (Figure 1C,E). WB analysis of heart tissues demonstrated a significant downregulation of PKP2 protein expression in the model groups (Figure 1D,F). These results indicate that HF may disrupt desmosomal structures, leading to the degradation of PKP2.

### 2.2 The Impact of *Pkp2* Knockout on Cardiac Function

**FIGURE 2:**
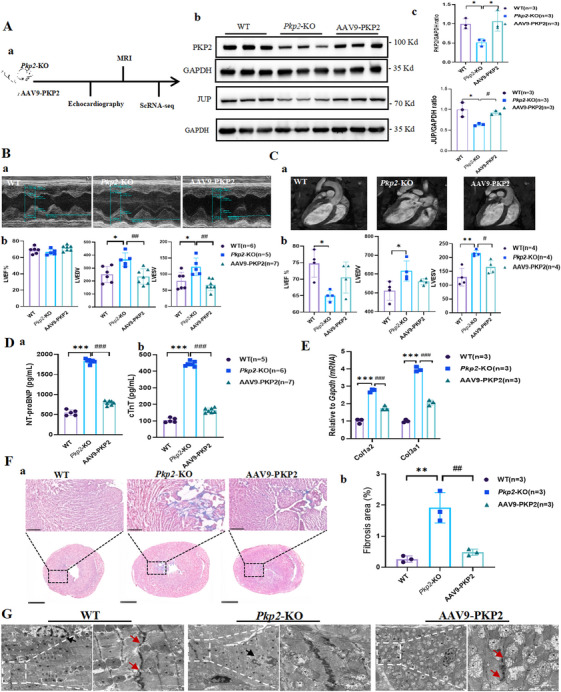
Construction of *Pkp2*‐knockout (*Pkp2*‐KO) rat model and validation of HF phenotype. (A) Expression of PKP2 protein and the desmosomal protein JUP in cardiac tissues of the wild‐type (WT) group, *Pkp2*‐KO group, and adeno‐associated virus serotype 9 (AAV9)‐PKP2 rescue group (*n* = 3). (B) Echocardiographic assessment of cardiac function in the control group, *Pkp2*‐KO group, and AAV9‐PKP2 rescue group (*n* = 6:5:7). (C) Small animal magnetic resonance imaging (MRI) evaluation of cardiac function in the control group, *Pkp2*‐KO group, and AAV9‐PKP2 rescue group (*n* = 4). (D) ELISA analysis of HF biomarkers NT‐proBNP and cTnT in serum from the control group, *Pkp2*‐KO group, and AAV9‐PKP2 rescue group (*n* = 5:6:7). (E) RT‐qPCR analysis of mRNA expression of collagen‐related genes (*Col1a2* and *Col3a1*) in cardiac tissues from the control group, *Pkp2*‐KO group, and AAV9‐PKP2 rescue group (*n* = 3). (F) Masson staining showing fibrosis in cardiac tissues of different groups and quantitative analysis results (*n* = 3). (G) Electron microscopy observation of desmosomal structures in cardiac tissues of different groups (*n* = 3). All data are presented as mean ± SEM. *p < 0.05, **p < 0.01 and ***p < 0.001.

To investigate the role of PKP2 in cardiac function, we performed *Pkp2* knockout in normal rats and conducted rescue therapy using AAV9‐PKP2. First, the efficiency of *Pkp2*‐KO was confirmed by WB analysis, which revealed a significant downregulation of the desmosomal protein JUP in the knockout group. Notably, AAV9‐PKP2 supplementation not only restored PKP2 expression but also increased JUP protein levels (Figure 2A). Subsequently, small animal magnetic resonance imaging (MRI) demonstrated that *Pkp2* knockout led to a significant reduction in LVEF and a marked increase in left ventricular end‐diastolic volume (LVEDV) and left ventricular end‐systolic volume (LVESV). These abnormalities were partially ameliorated by AAV9‐PKP2 supplementation (Figure 2B), a trend consistent with echocardiography results (Figure 2C). These findings suggest that *Pkp2* knockout impairs cardiac function, resulting in an HF phenotype.

Further analysis of serum biomarkers revealed a significant elevation in NT‐proBNP, a biomarker of HF, and cardiac troponin T (cTnT), an indicator of myocardial injury, in *Pkp2*‐KO rats. These levels were significantly reduced following AAV9‐PKP2 supplementation (Figure 2D), indicating that *Pkp2* knockout increases cardiac load and causes cardiomyocyte damage. Additionally, the expression of fibrosis‐related genes, including collagen type III alpha 1 chain (*Col3a1*) and collagen type I alpha 2 chain (*Col1a2*), was significantly upregulated in *Pkp2*‐KO rats and downregulated after PKP2 rescue therapy (Figure 2E). This trend was corroborated by a quantitative analysis of collagen fibers in Masson's trichrome‐stained cardiac tissues, suggesting that *Pkp2* knockout exacerbates myocardial fibrosis and contributes to HF progression (Figure 2F).

Finally, ultrastructural changes in desmosomal architecture were examined using electron microscopy (Figure 2G). *Pkp2* knockout resulted in irregular nuclear (N) morphology, severe nuclear membrane invagination, increased heterochromatin, and blurred local membrane structures. Intercalated discs (IDs) exhibited elongated dense regions with indistinct local structures, and no discernible desmosomes were observed. In contrast, AAV9‐PKP2 supplementation restored nuclear membrane integrity and revealed two distinct desmosomal structures. These results indicate that abnormalities in desmosomal structures disrupt intercellular connections, ultimately leading to the development of HF.

### 2.3 Long‐Term Safety Evaluation of AAV9‐PKP2 In Vivo

AAV9 demonstrates high in vivo safety but may elicit immune responses and systemic toxicity at high doses. To investigate the in vivo safety profile of AAV9, we collected liver, kidney, and lung tissues from rats 24 weeks after AAV9‐PKP2 injection and performed H&E staining. Results showed no significant inflammatory cell infiltration, tissue necrosis, or other notable pathological alterations in these critical organs (Figure S2A). At the same timepoint (24 weeks post‐injection), key serum biomarkers were analyzed: C‐reactive protein (CRP) for systemic inflammation, creatinine (Cr) for renal function, and alanine aminotransferase (ALT) for liver function. Compared to *Pkp2*‐KO group, the AAV9‐PKP2 group exhibited significantly reduced serum levels of CRP, Cr, and ALT (Figure S2B). These data indicate that the AAV9‐PKP2 vector caused no observable acute or subacute tissue damage in the examined organs, providing preliminary support for its safety profile.

### 2.4 Long‐Term Expression Study of AAV9 Vector in the Heart

To evaluate the metabolic kinetics and persistence of AAV9 vector expression in rat cardiac tissue, specific immunofluorescence zsGreen staining analysis was conducted on rat cardiac tissues at various time points (1, 2, 3, 4, 6, 8, 12, 24, and 48 weeks) following systemic administration of the AAV9 vector. The results demonstrated that a significant zsGreen expression signal with high fluorescence intensity could be detected in myocardial tissue as early as 6 weeks post‐injection, and this expression remained stable throughout the duration of the study (up to 48 weeks), with a clear positive signal still evident at the endpoint (Figure S3A). These findings indicate that the AAV9 vector is capable of achieving long‐term and stable transgenic expression in the context of heart‐targeted gene therapy, highlighting its potential application in the treatment of chronic cardiovascular diseases.

### 2.5 ScRNA‐Seq Analysis of Cardiac Cell Subpopulation Changes Following *Pkp2* Knockout

**FIGURE 3:**
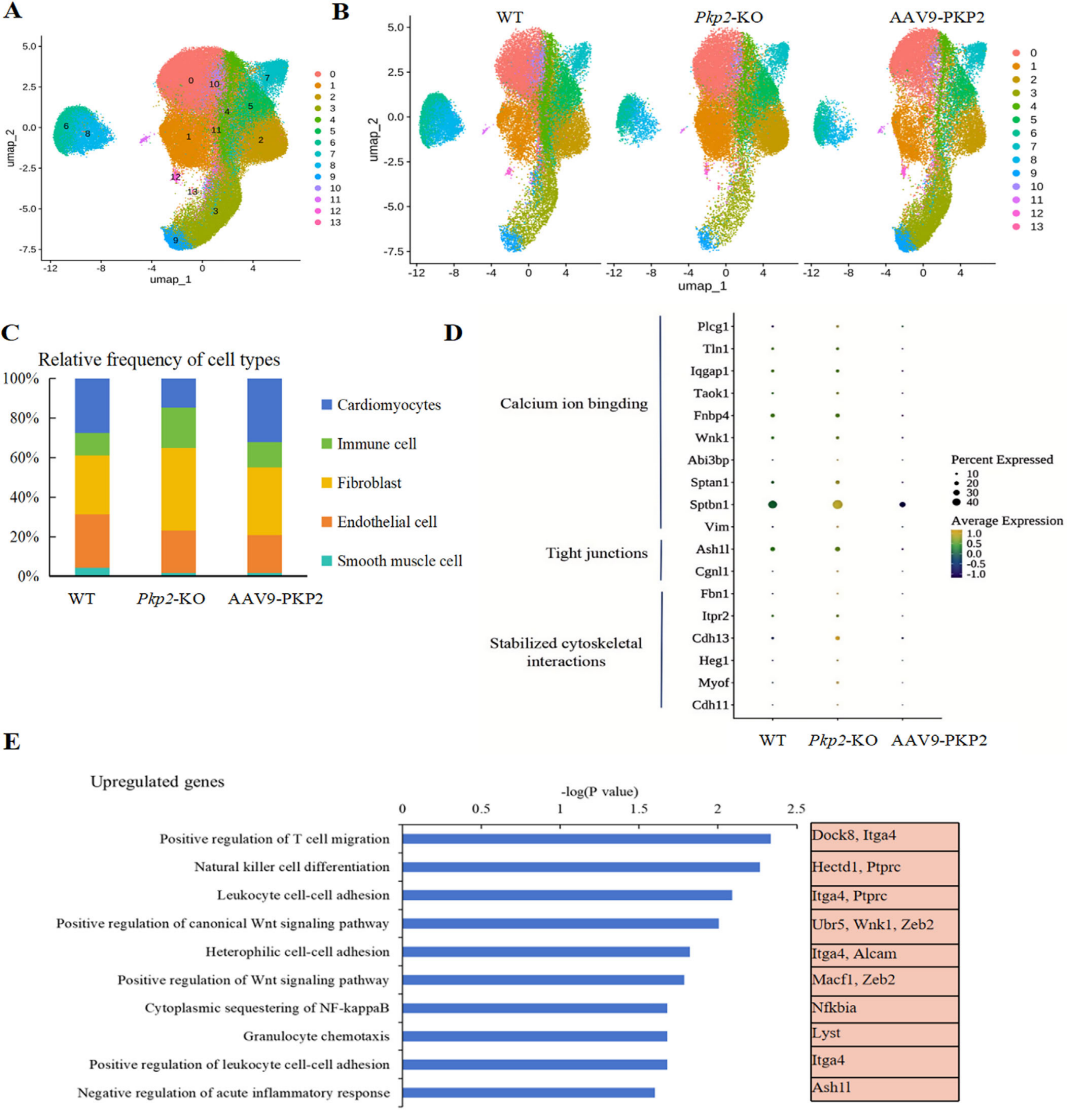
AAV9‐PKP2 therapy changed the cardiac cell landscape of HF. (A) Uniform manifold approximation and projection (UMAP) integrating 85113 single cells from WT, *Pkp2*‐KO, and AAV9‐PKP2 ventricles. Fourteen clusters are indicated by colors. (B) UMAP of cardiac colored by clusters in WT, *Pkp2*‐KO, and AAV9‐PKP2 (*n* = 3). (C) Bar plot of the percentage of cell type contributions in each scRNA‐seq sample. (D) Differential genes (up‐regulated in *Pkp2*‐KO) in cardiomyocytes are represented by dot plots. (E) Enrichment cluster analysis of significantly upregulated genes in immune cells of *Pkp2*‐KO group is shown.

We utilized the BD Rhapsody platform to perform scRNA‐seq for the classification of cardiac tissue cell subpopulations (Figures 3A,B, and S4A). Canonical correlation analysis (CCA) was employed for batch correction, followed by clustering analysis of the subpopulations. Unsupervised clustering revealed five distinct cell populations, including cardiomyocytes (marked by *Myh6, Tnnt2, Myl3*, and *Myl2*), endothelial cells (marked by *Ptprb, Nrp2*, and *Poglut2*), fibroblasts (marked by *Col3a1* and *Cdh11*), smooth muscle cells (marked by *Abcc9*), and immune cells (marked by protein tyrosine phosphatase receptor type C [*Ptprc*] and *S100a9*; Figure S4B,C). Compared to the WT and AAV9‐PKP2 groups, the *Pkp2*‐KO group exhibited an increase in fibroblasts and immune cells, alongside a reduction in cardiomyocytes (Figure 3C).

Previous studies have demonstrated that myocardial fibrosis is a process of cardiac interstitial remodeling characterized by the abnormal proliferation of CFs within myocardial tissue. For instance, during myocardial infarction, necrotic cardiomyocytes are replaced by collagenous scar tissue. Following cardiac injury, inflammatory signaling molecules are immediately upregulated [21, 22]. Our findings suggest that PKP2 may ameliorate fibrosis in HF patients by modulating the proportions of cardiomyocytes, fibroblasts, and immune cells in cardiac tissue.

To further elucidate the mechanisms underlying PKP2‐related therapeutic effects, we examined various cardiomyocyte subpopulations. By analyzing the scRNA‐seq datasets from WT, *Pkp2*‐KO, and AAV9‐PKP2 samples, we identified differentially expressed genes in the cardiomyocyte subpopulation. Compared to the WT and AAV9‐PKP2 groups, the *Pkp2*‐KO group exhibited upregulation of genes primarily associated with three categories (Figure 3D): calcium ion binding, tight junctions, and stabilizing cytoskeletal interactions. Conversely, downregulated genes in the *Pkp2*‐KO group (Figure S4D) were primarily related to sarcomere composition (*Tnnt2, Actc1, Myl2, Tpm1, Myl3*, and *Myh7*) and ischemia induction (*Nckap5* and *Vegfa*).

In the immune cell subpopulation, compared to the WT and AAV9‐PKP2 groups, the *Pkp2*‐KO group showed upregulation of genes enriched in several GO pathways (Figure 3E), including positive regulation of T‐cell migration (*Dock8* and *Itga4*), natural killer cell differentiation (*Hectd1* and *Ptprc)*, leukocyte cell–cell adhesion (*Itga4* and *Ptprc*), and positive regulation of the Wnt signaling pathway (*Ubr5, Wnk1*, and *Zeb2*), among others. These results indicate the critical role of PKP2 in regulating cardiac cell heterogeneity and provide insights into the molecular mechanisms underlying its protective effects in cardiac pathology.

### 2.6 Cell Subpopulation Analysis

**FIGURE 4:**
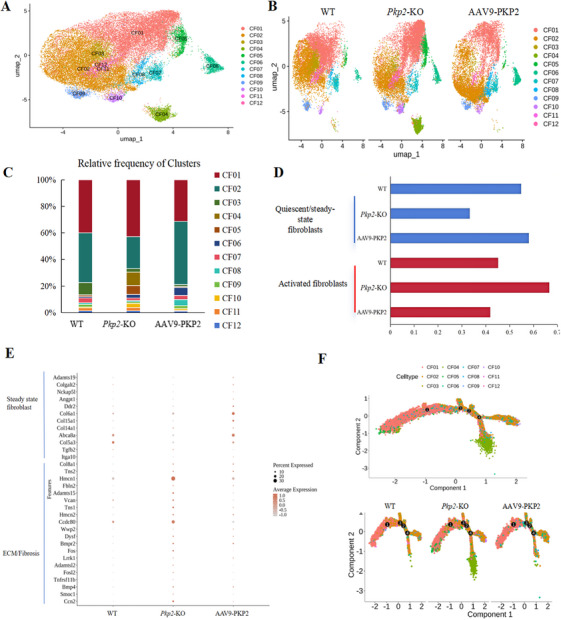
Single cell atlas of cardiac fibroblasts (CFs). (A) UMAP embedding 33413 CFs from WT, *Pkp2*‐KO, and AAV9‐PKP2 groups. Twelve clusters (CF01 through CF12) are identified. (B) UMAP of CFs colored by clusters in WT, *Pkp2*‐KO, and AAV9‐PKP2 groups (*n* = 3). (C) Bar plot of the percentage of CF cluster contributions in each single‐cell RNA sequencing (scRNA‐seq) sample. (D) Classification of fibroblasts in WT, *Pkp2*‐KO, and AAV9‐PKP2 groups was shown. (E) The signature genes from three groups of samples are represented by dot plots. The color and size of the dots indicate the relative average expression level in each population and the percentage of cells expressing the gene, respectively. (F) Trajectory analysis shows the evolutionary direction of all subpopulations of CFs; the evolutionary direction is from left to right.

We focused on the CF subpopulation to further explore the mechanisms underlying the anti‐fibrotic effects of PKP2 therapy. Unsupervised clustering revealed 12 distinct clusters within the CF subpopulation (Figure 4A–C). Among these, CF02, CF03, CF09, CF11, and CF12 were enriched with quiescent/steady‐state fibroblasts (marked by *Dpt* and *Gfpt2*) and extracellular matrix (ECM) degradation‐related genes (*Col5a3, Col1a1*, and *Col3a1*). CF01 (*Apoe* and *Clu*), CF04 (*Apoe*), and CF08 (*Igfbp3*) were classified as F‐WntX (fibroblast‐Wnt expressing) and F‐Trans (fibroblast‐transitory). F‐WntX fibroblasts are defined by high expression of Wnt pathway genes, while F‐Trans represents an intermediate state transitioning from quiescent/steady‐state fibroblasts to F‐WntX. CF10 (*Postn*) and CF05 (*Egr1* and *Fos)* were identified as F‐Act (fibroblast‐activated) and MFC (matrifibrocytes), expressing genes associated with activated fibroblasts, cartilage development, and ossification (Figures S5A,B and S6A).

Compared to the WT and AAV9‐PKP2 groups, the *Pkp2*‐KO group exhibited a reduced proportion of quiescent/steady‐state fibroblasts and an increased proportion of activated fibroblasts (Figure 4D). Differential gene expression analysis revealed that, compared to the WT group, the *Pkp2*‐KO group showed upregulation of multiple ECM and pro‐fibrotic genes (*Ccn2, Smoc1, Bmp4, Tnfrsf11b, Fosl2, Adamtsl2, Lrrk1, Fos, Bmpr2, Dysf, Wwp2, Ccdc80, Hmcn2, Tns1, Vcan, Adamts15, Fbln2, Hmcn1, Tns2*, and *Col8a1*), while genes associated with quiescent fibroblasts (*Itga10, Tgfb2, Col5a3, Abca8a, Col14a1, Col15a1, Col6a1, Ddr2, Angpt1, Nckap5l, Col5a3, Colgalt2*, and *Adamts19*) were downregulated (Figure 4E). Notably, these gene expression changes were reversed to normal levels following PKP2 supplementation, indicating a potential anti‐fibrotic effect of PKP2 therapy.

To further elucidate the dynamics and transitions of fibroblast subpopulations over time, we performed pseudotime analysis. The evolutionary trajectory of fibroblasts, moving from left to right, revealed a significant enrichment of the CF04 subpopulation in the later stages of the *Pkp2*‐KO group (Figures 4F and S6B). The CF04 subpopulation, classified as F‐Trans, further confirmed that *Pkp2* knockout leads to the activation of quiescent fibroblasts in the later stages. At the same time, PKP2 therapy can convert activated fibroblasts back to a quiescent state. These findings highlight the critical role of PKP2 in regulating fibroblast activation and provide insights into its therapeutic potential in mitigating cardiac fibrosis.

### 2.7 Analysis of Cell Communication

**FIGURE 5:**
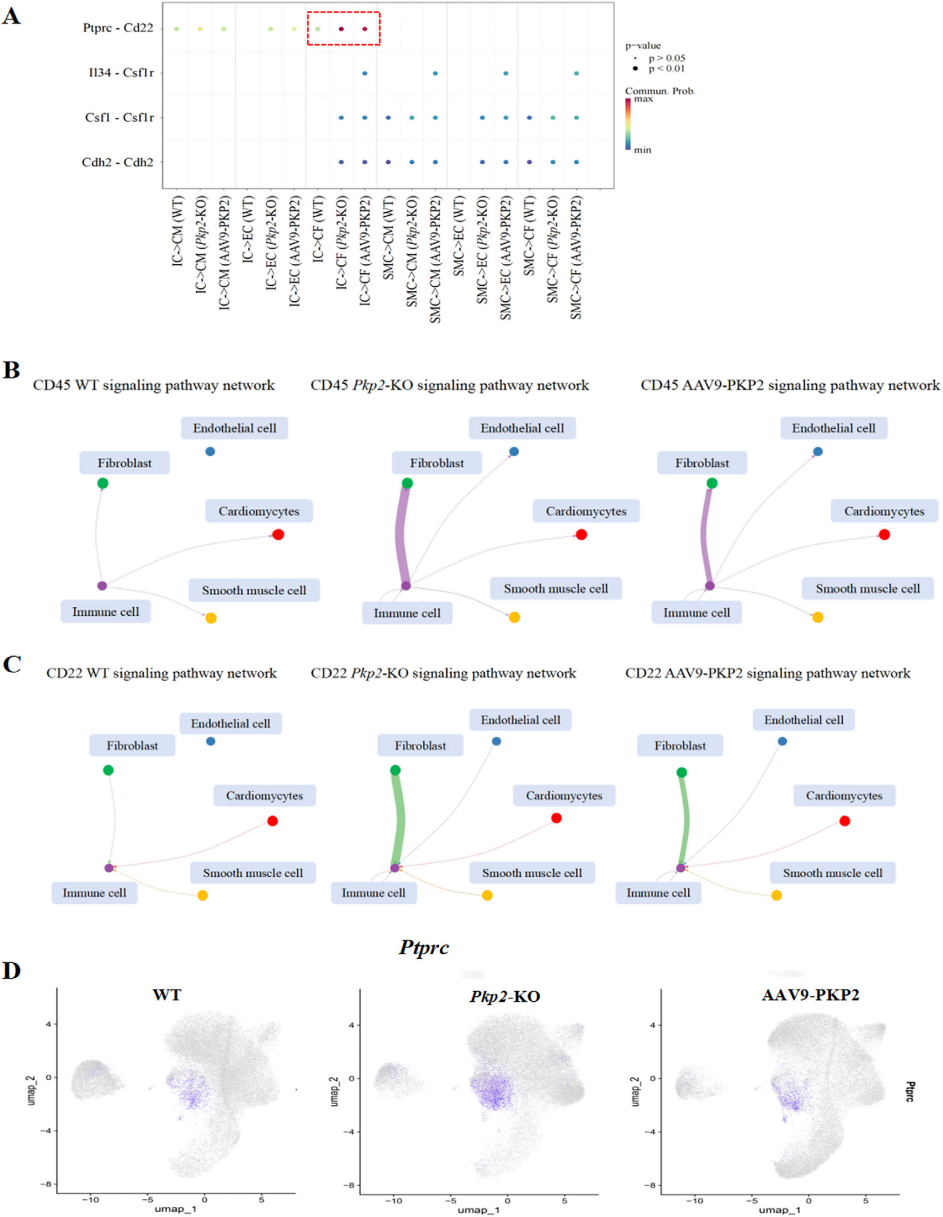
Interaction between immune cells and fibroblasts in HF disease. (A) The bubble diagram shows the differences between all the ligand‐receptor pairs in WT, *Pkp2*‐KO, and AAV9‐PKP2 groups. IC (immune cell), CM (cardiomyocytes), EC (endothelial cell), CF (fibroblast), SMC (smooth muscle cell). (B) CD45 signaling pathway network in WT, *Pkp2*‐KO, and AAV9‐PKP2 groups. (C) CD22 signaling pathway network in WT, *Pkp2*‐KO, and AAV9‐PKP2 groups. (D) Expression of protein tyrosine phosphatase receptor type C (*Ptprc*) as visualized on UMAP plots.

**FIGURE 6:**
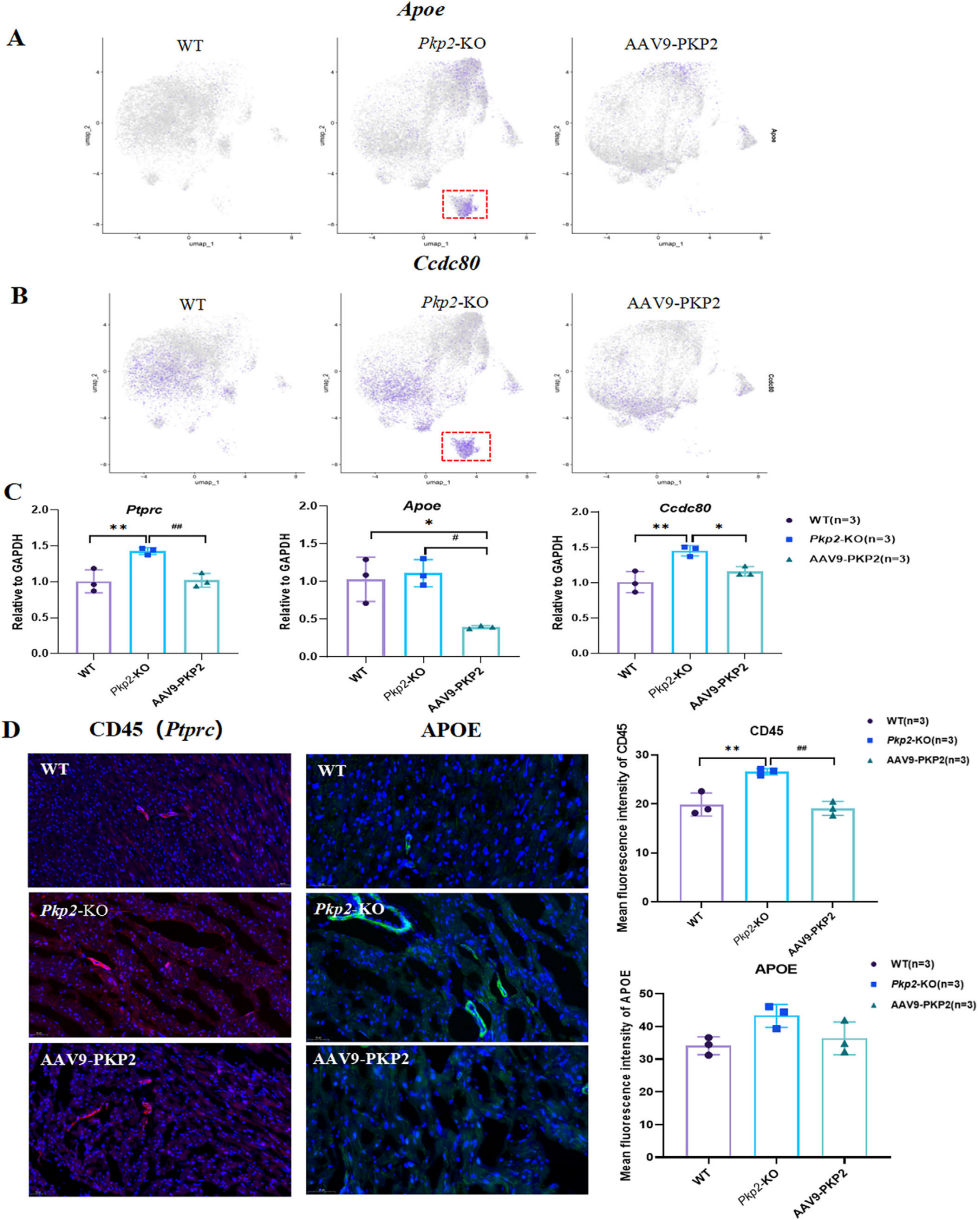
Expression verification of key genes in cell communication. (A) Expression of *Apoe* in fibroblast subgroup. (B) Expression of *Ccdc80* in fibroblast subgroup. (C) The mRNA expression of *Ptprc*, *Apoe*, and *Ccdc80* genes was detected by RT‐qPCR (*n* = 3). (D) The expression of CD45, APOE in cardiac tissue was detected by immunofluorescence (*n* = 3, scale bar: 100 µm). All data are presented as mean ± SEM. *p < 0.05, **p < 0.01, and ***p < 0.001.

We hypothesized that the transition between quiescent and activated fibroblasts might be associated with immune cells. To investigate this, we employed the CellChat algorithm to analyze intercellular interactions among cell subpopulations (Figure S6C,D). The analysis revealed predictable interactions between immune cells expressing *Ptprc* and fibroblasts expressing CD22 (Figure 5A). Compared to the WT and AAV9‐PKP2 groups, the *Pkp2*‐KO group exhibited stronger *Ptprc*‐CD22 communication (Figure 5B,C). Notably, *Ptprc* was highly expressed in the *Pkp2*‐KO group (Figure 5D). CD22 is known to inhibit B‐cell receptor‐induced signaling, characterized by the suppression of calcium mobilization and cell activation [23]. The CF04 subpopulation, identified as an intermediate state of activated fibroblasts, showed high expression of *Apoe* and *Ccdc80* (Figure 6A,B). We speculated that *Ptprc* might influence *Apoe* and *Ccdc80* to mediate the transition and identity shift of fibroblasts. To further validate the cell communication results, we measured the mRNA expression levels of *Apoe*, *Ccdc80*, and *Ptprc* in cardiac tissues. The results demonstrated that *Ccdc80* and *Ptprc* were significantly upregulated in the *Pkp2*‐KO group and downregulated following treatment (Figure 6C), consistent with the immunofluorescence staining of cardiac tissue sections (Figure 6D). In contrast, *Apoe* expression did not show a significant increase in the *Pkp2*‐KO group but was markedly reduced after treatment (Figure 6C). In addition, we also detected the expression of CD45 and APOE in hypoxia‐damaged cardiac organoids. The results showed that CD45 and APOE were significantly increased in the model group (Figure S1C), further supporting the single‐cell sequencing results of rats. These findings suggest that CD45 may play a critical role in regulating fibroblast activation through interactions with APOE and CCDC80, providing further insights into the mechanisms underlying PKP2‐mediated anti‐fibrotic effects.

### 2.8 Expression of Candidate Target Genes in Mouse Model of HF

**FIGURE 7:**
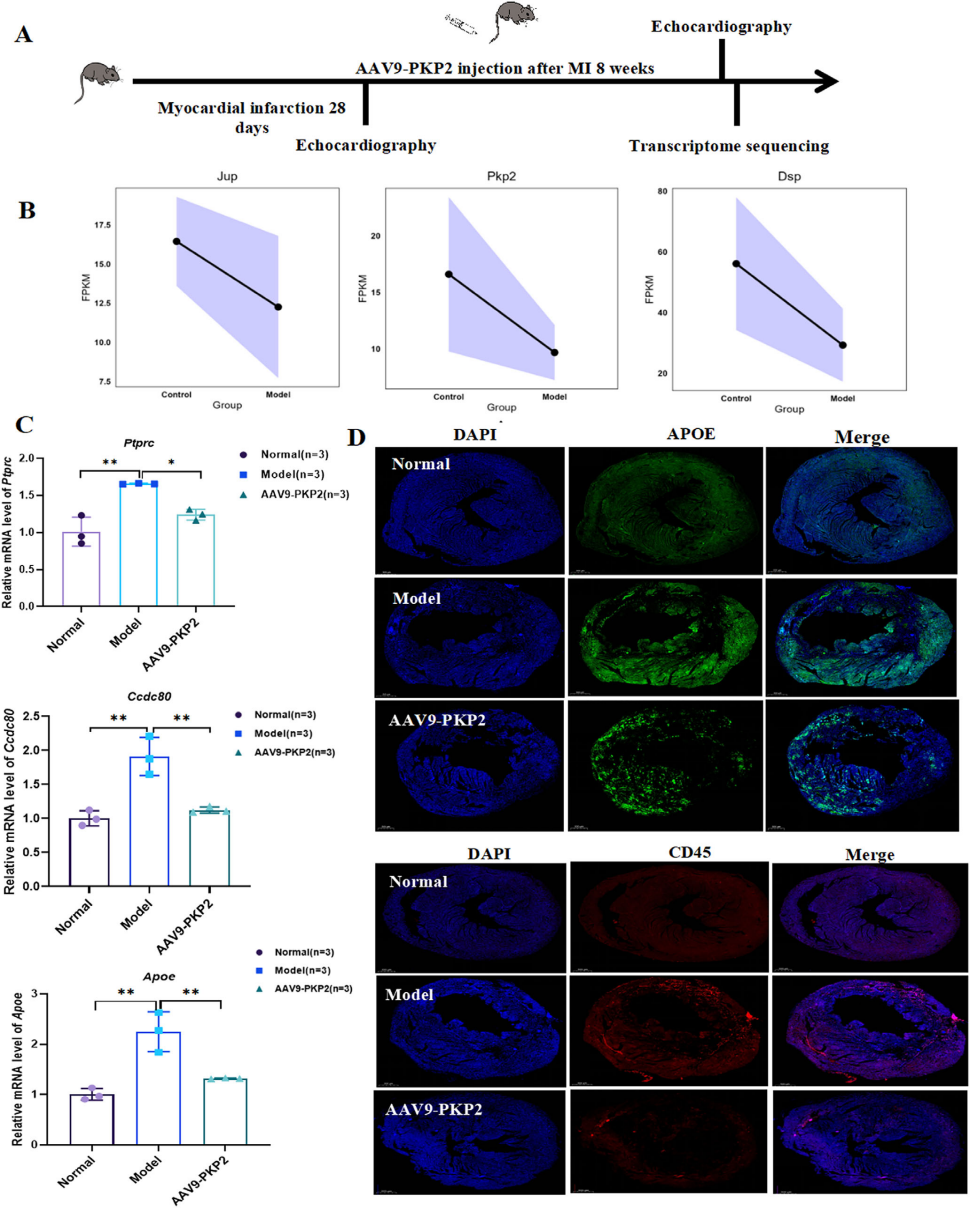
Transcriptome sequencing expression results and experimental validation in HF mice. (A) Schematic diagram of the construction of HF mice. (B) Transcriptome sequencing expression results of desmosomal genes, including *Pkp2*, *Jup*, and *Dsp* genes in HF mice (FPKM is an indicator in transcriptome sequencing that quantifies gene expression levels and facilitates comparisons between different genes and samples by normalizing gene length and sequencing depth). (C) RT‐qPCR analysis of *Ptprc, Apoe*, and *Ccdc80* gene expression in cardiac tissues of HF mice (*n* = 3). (D) Results of CD45 and APOE immunofluorescence staining in mouse heart tissue (scale bar: 500 µm). All data are presented as mean ± SEM. *p < 0.05, **p < 0.01, and ***p < 0.001.

Transcriptomic sequencing was performed in the HF mouse model (Figure 7A). The results revealed a downregulation trend in the expression of *Pkp2* and related desmosomal genes, *Jup* and *Dsp*, in the model group (Figure 7B), consistent with our previous findings in *Pkp2*‐KO rats. Further analysis of mRNA expression levels of *Apoe*, *Ccdc80*, and *Ptprc* in mouse cardiac tissues demonstrated that *Apoe* and *Ptprc* were significantly upregulated following *Pkp2* knockout and downregulated after treatment (Figure 7C). Immunofluorescence staining of cardiac tissue sections with CD45 and APOE also showed the same result (Figure 7D). These findings suggest a close association between PKP2 and APOE/CD45, highlighting their potential roles in the pathogenesis and treatment of HF.

### 2.9 Validation of AAV9‐PKP2 Replenishment Therapy in HF Mice

**FIGURE 8:**
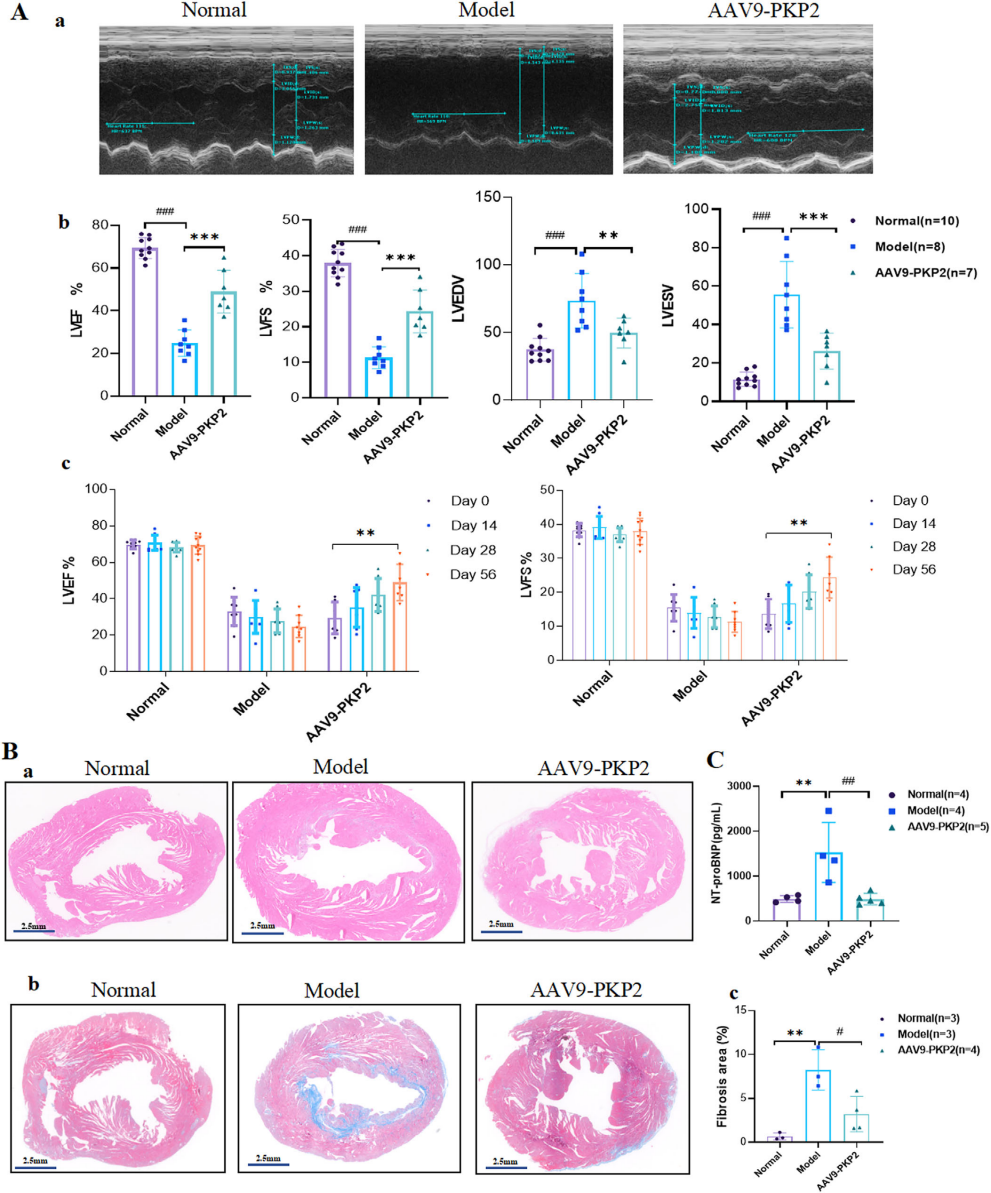
AAV9‐PKP2 improves cardiac function and reduces fibrosis in HF mice. (A) Echocardiographic assessment of cardiac function‐related parameters (LVEF, LVFS, LVEDV, LVESV) in the normal, model group, and AAV9‐PKP2 groups (*n* = 10:8:7). (B) HE and Masson staining showing pathological changes in cardiac tissues of the normal, model, and AAV9‐PKP2 groups (*n* = 3; scale bar: 2.5 mm). (C) ELISA analysis of HF biomarkers NT‐proBNP in serum from the normal, model, and AAV9‐PKP2 groups (*n* = 4:4:5). All data are presented as mean ± SEM. *p < 0.05, **p < 0.01, and ***p < 0.001.

Based on these findings, we administered AAV9‐PKP2 gene supplementation therapy to HF mice. Echocardiographic results eight weeks post‐treatment revealed that, compared to the model group, the AAV9‐PKP2 supplementation group exhibited a significant increase in LVEF and LVFS, along with a significant reduction in left ventricular LVEDV and LVESV (Figure 8A). These results demonstrate that AAV9‐PKP2 supplementation therapy significantly improves cardiac function and attenuates ventricular remodeling in HF mice. HE staining showed fibrosis and mild chronic inflammatory cell infiltration in specific regions of the left ventricle in the model group, while inflammation was alleviated in the AAV9‐PKP2 supplementation group (Figure 8B). Masson staining further revealed a significant increase in fibrosis in the model group, which was suppressed in the AAV9‐PKP2 supplementation group, indicating inhibition of the fibrotic process induced by HF (Figure 8B). In addition, we also assessed the expression of NT‐proBNP, a biomarker of HF, in the serum of mice. The results indicated that NT‐proBNP levels in the AAV9‐PKP2 group were significantly reduced, compared to those in the model group (Figure 8C). Similarly, liver, kidney, and lung tissues collected from mice 16 weeks after AAV9‐PKP2 injection revealed no significant hepatorenal or pulmonary toxicity in HE staining, except for minor vascular congestion attributable to myocardial infarction modeling (Figure S2C). Collectively, these findings suggest that AAV9‐PKP2 supplementation therapy has a beneficial therapeutic effect in HF mice, highlighting its potential for clinical application in mitigating HF progression.

## 3 Discussion

### 3.1 PKP2 Dysregulation: A Molecular Bridge Linking ARVC to HF

PKP2, a cardinal structural component of cardiomyocyte IDs, has been extensively characterized as a genetic determinant in ARVC, with loss‐of‐function mutations accounting for approximately 40% of familial ARVC cases [24, 25]. Our study extends this paradigm by demonstrating significant PKP2 downregulation across multiple post‐myocardial infarction HF models, suggesting its potential role as a molecular mediator in acquired cardiac pathologies beyond its established genetic associations. In addition, the expression level of the *pkp2* gene was downregulated in the HF group (normal group: 137.776 ± 24.63478; HF group: 125.8 ± 10.82799), based on the gene expression matrix from the Gene Expression Omnibus (GEO) database (accession number GSE120852). The observed phenotypic convergence between ARVC and HF, despite their distinct etiological origins (monogenic mutations vs. ischemic/pressure overload), reveals fundamental mechanistic commonalities. Both conditions exhibit progressive myocardial remodeling characterized by fibrofatty replacement and electromechanical dysfunction, with advanced ARVC frequently progressing to biventricular failure that clinically mirrors end‐stage HF [26]. This pathophysiological overlap suggests that PKP2 dysfunction may represent a “final common pathway” mediating adverse cardiac remodeling across diverse disease states.

Our findings position PKP2 as a pivotal molecular node integrating genetic and acquired cardiomyopathy pathways. The protein's expression dynamics and functional status may serve as a quantitative biomarker for myocardial remodeling severity, offering potential utility in both diagnostic stratification and therapeutic monitoring. This dual role of PKP2 as both a genetic susceptibility factor and an acquired remodeling mediator provides novel mechanistic insights into the shared molecular underpinnings of ARVC and HF progression.

### 3.2 Omics Sequencing Combined With scRNA‐Seq Reveals Cellular Subpopulation Trends and Omics Correction as Markers of Effective Gene Supplementation Therapy

Using high‐throughput omics sequencing and scRNA‐seq technologies, we conducted an in‐depth analysis of cellular subpopulation changes and omics profiles in cardiac tissues of rats following *Pkp2* knockout and subsequent rescue therapy. The results demonstrated that post‐treatment, the overall gene expression profiles normalized, along with positive changes on the proportions and functions of specific cell subpopulations (e.g., cardiomyocytes, fibroblasts). This suggests that gene supplementation not only directly restores the expression of the defective gene but may also promote overall cardiac tissue repair by modulating intercellular interactions and the microenvironment. ScRNA‐seq further revealed shifts in cellular heterogeneity before and after treatment, providing more precise cytological markers for evaluating therapeutic efficacy. These findings not only enhance our understanding of the pathophysiological mechanisms underlying HF but also offer new perspectives and tools for assessing the effectiveness of future gene therapies [27]. By integrating omics and single‐cell approaches, this study underscores the potential of multi‐omics strategies in guiding and optimizing gene‐based therapeutic interventions.

### 3.3 Discovery of Novel Key Molecular Mechanisms

During the in‐depth exploration of *Pkp2* deficiency in the pathogenesis of ARVC, we uncovered novel molecular mechanisms that may be directly regulated by PKP2 and contribute to disease progression. For instance, certain genes, such as *Ptprc* and *Apoe*, were aberrantly activated or suppressed in *Pkp2*‐KO rats. These genes are closely associated with cardiomyocyte proliferation, differentiation, apoptosis, and fibrosis.

One such gene, protein tyrosine *Ptprc*, also known as CD45, is a transmembrane glycoprotein expressed on almost all hematopoietic cells except mature erythrocytes [28]. It serves as a critical regulator of T‐cell and B‐cell antigen receptor‐mediated activation [29]. Studies have shown that the development and progression of hepatic fibrosis are accompanied by inflammatory injury. Transcriptomic sequencing analysis of hepatic fibrosis rats revealed that the immune gene *Ptprc* may play a significant role in fibrosis formation and inflammatory damage, as validated by qPCR [30]. Additionally, *Ptprc* was identified as a potential common gene in renal fibrosis across three different types of advanced chronic kidney disease (CKD) based on tissue biopsy results [31].

In addition to *Ptprc*, another key gene, apolipoprotein E (*Apoe*), exhibited distinct changes in single‐cell sequencing. *Apoe* is best known for its roles in the central nervous system (e.g., Alzheimer's disease) and atherosclerosis [32]. As a component of low‐density lipoprotein (LDL) and high‐density lipoprotein (HDL), Apoe mediates reverse cholesterol transport from peripheral tissues, including the heart, to the liver, maintaining lipid homeostasis in cardiomyocytes [33]. Moreover, APOE4 carriers exhibit elevated plasma cholesterol levels, which are associated with an increased risk of atherosclerosis and ischemic heart disease [34]. In our study, the activation of *Ptprc* and *Apoe* was confirmed, and single‐cell sequencing results indicated abnormal activation of fibroblasts in the *Pkp2*‐KO group. Furthermore, predictable interactions were observed between immune cells expressing *Ptprc* and fibroblasts expressing CD22. We hypothesize that the therapeutic effect of AAV9‐PKP2 may involve regulating the interaction between CD45 and CD22, thereby interfering with fibrosis formation post‐HF through CCDC80 and APOE.

Collectively, these discoveries highlight the potential roles of *Ptprc* and *Apoe* in the pathogenesis of ARVC and HF, providing new insights into the molecular mechanisms underlying PKP2‐related cardiac dysfunction and fibrosis. These findings may pave the way for novel therapeutic strategies targeting these pathways.

### 3.4 Limitations

Although our data clearly established the causal relationship between the down‐regulation of PKP2 protein, there are still certain deficiencies. The PKP2‐CD45 regulatory axis is not the central focus of this study, it is of importance to investigate the mechanistic role of CD45 in reversing cardioprotective effects in our study setting, the next, our group will focus on this mechanistic issue. Does the PKP2 protein directly influence the transcriptional regulation of CD45, or is it regulated indirectly through specific signaling pathways? Alternatively, could its effect be mediated by altering mRNA stability or protein degradation? These issues need to be clarified in future research. Furthermore, an in‐depth exploration of single‐cell data is warranted. This should involve a focus on specific immune cell subsets exhibiting significantly elevated levels of CD45, along with an analysis of their differentially expressed genes and alterations in cellular communication. The goal would be to identify potential regulatory factors or indirect regulatory pathways involved. Such investigations will provide a robust theoretical foundation and precise targets for developing novel strategies for HF immunotherapy that leverage this regulatory axis.

In summary, this study identified the downregulation of PKP2 in HF models and through single‐cell sequencing of *Pkp2*‐KO rats, revealed therapy‐related cytological and omics markers. Additionally, novel potential molecular mechanisms were uncovered, providing new insights and directions for the treatment of HF. These findings not only enhance our understanding of the pathological mechanisms underlying HF but also offer a foundation for the development of targeted therapeutic strategies.

## 4 Methods

### 4.1 Construction of HF Model of Heart Organoids

When the iPSCs recovered and reached an adaptive growth state, organoid differentiation was initiated. The cells were dissociated using a gentle digestion solution, followed by cell counting and seeding to ensure approximately 10,000 cells per well in 96‐well plates. On Day 2 of differentiation, 100 µL of mTESR medium was added to each well. On Day 3, the medium was replaced with Differentiation Medium A containing CHIR99021 (MCE, USA; HY‐10182), BMP4 (MCE, USA; HY‐P7007), LY294002 (MCE, USA; HY‐10108), Activin A (MCE, USA; HY‐P70311), and insulin (Pricella, China; PB180432), followed by a 48‐h incubation. Subsequently, the medium was changed to Differentiation Medium B containing BMP4, IWR1 (MCE, USA; HY‐12238), retinoic acid (MCE, USA; HY‐14649), FGF2 (MCE, USA; HY‐P7004), and insulin for 4 days. This was followed by replacement with Medium C supplemented with FGF2 for 5 days. Finally, the cultures were maintained in Maturation Medium M, containing insulin until full maturation. All media were refreshed daily throughout the differentiation process. After successful organoid differentiation, the culture medium was replaced with sugar‐free and serum‐free DMEM medium (Gibco, USA; 11966025). The samples were then placed in a tri‐gas incubator with oxygen concentration adjusted to 5% for continuous hypoxia treatment over 24 h. Then test relevant indicators to evaluate the effectiveness of mold manufacturing.

### 4.2 Animals and Materials

The *Pkp2*‐KO rat as well as their WT littermates were obtained from Prof. Wang Xin (East China Normal University, Shanghai, China). All rats were fed in the Laboratory Animal Center of Putuo Hospital, Shanghai University of Traditional Chinese Medicine. The animals are housed in SPF‐rated spaces with plenty of food and water. CMV‐carrying rat/mouse desmosomal protein PKP2 (AAV9‐PKP2) was produced by Jiman Biotechnology Co. Ltd., Shanghai, China. AAV9‐PKP2 was injected directly into the tail vein of 6‐week‐old rats (1E12vg per 100 g body weight in rats). After 4 weeks of MI modeling in 8‐week‐old mice, AAV9‐PKP2 was directly injected into the tail vein (5E11vg/mouse). Cardiac tissues were collected after different injection times, and the transduction efficiency of AAV in vivo was evaluated by the immunofluorescence method.

Construction of an HF model following myocardial infarction in mice: Forty SPF (specific pathogen‐free) grade male C57BL/6J mice (8 weeks old, weighing 20–25 g) were selected. All mice were adaptively fed before the formal experiment. The mice were divided into two groups, namely, the sham operation group and the HF group. During the modeling operation, 3%–5% isoflurane was used to perform respiratory anesthesia on mice. The mice were fixed in the supine position on the mouse board. The skin on the chest of the mice was disinfected with iodophor, and the hair at the surgical site was removed. Then, the skin on the chest and abdomen was routinely disinfected. After tracheal intubation, it was connected to a small animal ventilator. Thoracotomy was performed in the left precordial area near the fourth intercostal space. Ligation of the left anterior descending coronary artery was carried out in mice of the HF group between the pulmonary artery cone and the lower edge of the left atrial appendage, approximately 2–3 mm from the root of the aorta. The sham operation group only threaded but did not ligate. On the 28th day after the operation, all the mice that underwent echocardiographic cardiac function examination were screened for successful modeling according to LVEF < 50%.

For rat myocardial infarction surgical modeling, ligation of the left anterior descending coronary artery was performed. Echocardiography was conducted 28 days post‐operation to assess cardiac function, with an LVEF < 50% serving as the criterion for successful model establishment.

### 4.3 MRI

MRI scans of rats were performed using a BioSpec 94/30 USR (Bruker BioSpin MRI GMBH, Germany). The rats were placed in the corresponding grooves of the scanning bed in a prone position, and anesthetics were continuously delivered using a dedicated mask. Meanwhile, the rats were fixed with a specially designed bracket or fixation device. Electrodes for electrocardiogram (ECG) were inserted into the limbs of the rats, and a respiratory sensor was placed to monitor the ECG and respiratory status of the rats. The coil was connected, the scanning parameters were set, and the standard short‐axis position of the left ventricle of the heart was determined through shimming and positioning scans. Scans were conducted layer by layer from the apex to the base of the heart to obtain dynamic images of the rat heart.

### 4.4 Echocardiography

All rats were treated with hair removal in the chest and left axilla, and the rats were scanned with a small animal echocardiograph. The ultrasonic coupling agent was evenly applied on the chest of the rat, the ultrasonic probe was placed on the chest of the rat, and the angle and position of the probe were adjusted to obtain clear images of the left ventricular long axis and left ventricular short axis. In general, the probe should point to the upper right and the depth should be adjusted to 2.0–2.5 cm. Under the guidance of two‐dimensional images, M‐shaped curves are taken and measured. At the same time, Doppler blood flow spectrum images can be collected to evaluate the blood flow of each valve opening. Left ventricular end‐diastolic diameter (LVDd) and end‐systolic diameter (LVDs) were measured based on ultrasound images, and left ventricular ejection fraction (EF) and brachyaxis shortening rate (FS) were calculated.

### 4.5 scRNA‐Seq

The heart tissue was cut into small pieces of about 1–2 mm^3^ and digested in the SoloTM dissociation kit (Sinotech Genomics, JZ‐SC‐58201) at 37°C for 30–60 min. The enzyme digestion was then terminated using excess RPMI‐1640 medium, and the cells were filtered through a 40‐µm cell screen. The single‐cell solution is stored on ice before being loaded onto the BD Rhapsody vector for single‐cell transcriptome capture. Then, single‐cell transcriptome capture, library construction, and sequencing were performed. First, the cells were stained using two fluorescent dyes, Calcein AM and Draq7, for precise determination of cell concentration and viability by the BD Rhapsody scanner (BD Biosciences). The cells were loaded into the BD Rhapsody microporous vector. The overloaded cells then capture magnetic beads to ensure that almost every microhole contains a magnetic bead, and the excess magnetic beads are subsequently flushed out of the carrier. After lysis of cells using lysis buffers, the cells are recovered and cleaned to capture magnetic beads, which are then reverse‐transcribed. The single‐cell transcriptome captured by the microbeads is converted into a cDNA library containing cell tags and unique molecular identifier (UMI) information. All procedures were performed in strict accordance with the manufacturer's protocol, using the BD Rhapsody cDNA kit (BD Biosciences, Product No. 633773) and the BD Rhapsody Targeted mRNA and AbSeq Amplification Kit (BD Biosciences, Product No. 633773). Product No. 633801) was carried out. All libraries were sequenced on the NovaSeq platform (Illumina) in PE150 mode (pin‐end, 150 bp per read).

### 4.6 Pathological Staining

The rat heart tissue was taken into the embedding box, and the tissue was immersed in an OCT embedding agent and frozen in the −80°C refrigerator. The embedded tissue was put into the frozen microtome and cut into 5‐µm slices. The slices were flat and pasted on clean slides. APOE (Abcam, ab183597, 1:500), CD45 (Abcam, ab10558, 1:200), α‐SMA (Abcam, ab5694, 1:200), cTnT (Abcam, ab209813, 1:100), CD31 (Santa Cruz Biotechnology, sc‐376764, 1:200), MYH7 (Servicebio, GB151857, 1:1:300), α‐actinin (Servicebio, GB115740, 1:200).

### 4.7 WB

Tissue and whole‐cell protein extracts were collected using a radioimmunoprecipitation (RIPA) lysis buffer (Beyotime, China; P0013B). A significant volume of protein was isolated by 10% sodium dodecyl sulphate‐polyacrylamide gel electrophoresis (SDS‐PAGE) and transferred to a 0.22‐µm polyvinylidene fluoride (PVDF) membrane (Millipore, St. Louis, MO, USA; IPVH00010). The membrane was incubated with 5% skimmed milk (Sigma‐Aldrich, Newark, NJ, USA) for 2 h, with histone PKP2 (Satan Cruz, USA; sc‐393711, 1:500), JUP (Abcam, UK; ab184919, 1:1000), CD45 (Abcam, UK; ab10558, 1:500), APOE (Abcam, UK; ab183597, 1:2000) and the antibodies were incubated at 4°C for 12 h. The membrane was then combined with the corresponding secondary antibody (CST, USA; 7074S and 7076S, 1:1000) at 25°C for 2 h. Finally, an enhanced chemiluminescence (ECL) Western Blotting test kit (Bio‐Rad, USA; 1705061) was used to detect immunoblotting. WB data were analyzed by Image‐Pro Plus 6.0 software (Tianneng, China).

### 4.8 RT‐qPCR

Total RNA was isolated from frozen cardiac tissue with RNAiso Plus (TaKaRa, Japan; 9109). cDNA was synthesized with a Takara reverse transcription kit (Takara, Japan; RR047A), and real‐time quantitative PCR was performed with TB Green chimeric fluorescence assay (Takara, Japan; RR420A) with specific primers described in Table S1. The expression levels of specific genes were normalized to the GAPDH gene.

### 4.9 Transcriptome Sequencing of Mouse Cardiac Tissue

RNA was extracted from mouse heart tissue, followed by assessment of RNA quality and concentration. After passing quality control, eukaryotic mRNA was enriched using magnetic beads with Oligo (dT). Fragmentation buffer was then added to randomly fragment the mRNA. Using the fragmented mRNA as a template, the first strand of cDNA was synthesized with random hexamers. Subsequently, the second cDNA strand was synthesized by adding buffer, dNTPs, and DNA polymerase I. Double‐stranded cDNA was purified using AMPure XP beads. The purified double‐stranded cDNA underwent end repair, addition of an “A” tail, and ligation of sequencing adapters. Fragment size selection was performed using AMPure XP beads, followed by PCR amplification to generate the final cDNA library. After library quality inspection, qualified libraries were pooled according to the target sequencing data volume and subjected to sequencing.

### 4.10 Statistical Analysis

A. Experimental data: Statistical analysis used SPSS 26 software. Results were expressed as the mean ± standard deviation (x ± s). Two groups comparison using a *t*‐test, and comparing multiple groups using one‐way ANOVA. *p* < 0.05 was regarded as statistically significant. Normality tests were conducted before applying the parameter statistical test.

B. Sequencing data: The raw sequencing data obtained from the cDNA library underwent comprehensive processing via the BD Rhapsody full transcriptome analysis pipeline (version 1.8). This protocol encompasses several critical steps: filtering based on read quality, reading annotations, assigning molecular annotations, identifying candidate cells, and ultimately generating single‐cell expression matrices. For all resultant output files, subsequent analyses were conducted utilizing UMI count matrices, which detailed the expression levels of each gene within individual cells. Notably, the BD process leveraged the Genome Reference Consortium Human Genome Build 38 (GRCh38) as the reference genome.

Analysis of scRNA‐seq data: Seurat software was utilized for subsequent clustering analysis and visualization. Initially, the gene expression matrix of each sample was imported and transformed into a Seurat object. In subsequent analyses, cells with a mitochondrial UMI ratio exceeding 35% or containing fewer than 50 genes were excluded. Following logarithmic normalization based on the total UMI count per cell, data scaling was performed relative to the UMI number. Principal component analysis (PCA) was conducted using the top 2000 highly variable features. Clustering was performed at a resolution of 0.5, and unified manifold approximation and projection (UMAP) was employed for data visualization. Feature plots, violin plots, and heatmaps were generated to illustrate the expression patterns of specific genes within each cluster.

Cell annotation: The FindAllMarkers function combined with the Wilcoxon test was used to calculate the specific markers for each cell cluster under the following criteria: log2 fold change > 0.25; min. Pct > 0.25. Referencing Cell Taxonomy (https://ngdc.cncb.ac.cn/celltaxonomy/) and Cell Marker (http://117.50.127.228/CellMarker/) helped us with a lot of the transcriptome data for each cell cluster annotation type.

C. Pseudo‐time trajectory analysis: After cell type annotation, single‐cell pseudo‐time analysis was performed using Monocle2 and DDR‐Tree dimensionality reduction methods. The single‐cell pseudo‐time analysis used the default parameters. In a nutshell, a Monocle object is first created from the presentation matrix and metadata information stored in the Seurat object. In the feature selection process, the Seurat cluster's top marker genes were set as sequencing genes for downstream analysis. The batch effect is also eliminated during dimensionality reduction. The results of pseudo‐time analysis are shown by using the trajectory diagram.

D. Cell communication: Cell Chat uses the gene expression data of cells as input, combined with the interaction of ligand receptors and their cofactors to simulate intercellular communication. Cell interactions are shown through heat maps and network maps.

## Author Contributions

Z.L. and X.W. conceptualized the study. L.X. created the methodology. H.Z. performed the formal analysis. X.D., X.Z., N.Y., S.H., X.J., and T.L. did the investigation. X.D. wrote the original draft. All authors wrote, reviewed, and edited the manuscript. It is confirmed that all authors have read and approved the final manuscript.

## Ethics Statement

All animal procedures were approved by the Institutional Animal Care and Use Committee (IACUC) (Animal Ethics Approval Number: DWEC‐A‐22024‐02‐2‐72).All experimental protocols on patients were approved by the Ethics Committee of Putuo Hospital, Shanghai University of Traditional Chinese Medicine (reference number: PTEC‐A‐2022‐13 (S) ‐1), and were conducted in accordance with the ethical standards of the Declaration of Helsinki.

## Conflicts of Interest

Author Zhen Qi is an employee of Neocellmed Co. Ltd. but has no potential relevant financial or non‐financial interests to disclose. The authors declare no conflcits of interest.

## Supporting information




**FIGURE S1**: Construction of cardiac organoid model. (A) Morphological images of cardiac organoids before and after hypoxia. (B) Cardiac signature maker (cTnT, CD31, αSMA, MYH7 and α‐actinin) immunofluorescence staining image of cardiac organoid. (C) The expression of CD45 and APOE proteins in hypoxia‐damaged cardiac organoids (n = 3). All data are presented as mean ± SEM. *p < 0.05, **p < 0.01, and ***p < 0.001.
**FIGURE S2**: Safety evaluation and metabolic level of AAV9‐PKP2 in vivo. (A) HE pathological staining of lung, liver, and kidney tissues of different groups of rats (*n* = 3). (B) Results of serum safety indexes (CRP, CR, ALT) in different groups (*n* = 5:7:7). (C) HE pathological staining of lung, liver, and kidney tissues of different groups of mice (*n* = 3). All data are presented as mean ± SEM. *p < 0.05, **p < 0.01, and ***p < 0.001.
**FIGURE S3**: Long‐term expression study of AAV9 vector in the heart. (A) Cardiac immunofluorescence staining after AAV9‐zsgreen injection in rats at different times (*n* = 3).
**FIGURE S4**: Characterization of cell cluster contributions and differential expression in scRNA‐seq Analysis. (A) Bar plot of the percentage of cluster contributions in each scRNA‐seq sample. (B) Dot plot visualization of top marker genes used to identify clusters. The color and size of the dots indicate the relative average expression level in each population and the percentage of cells expressing the gene, respectively. (C) Fraction of cell types. (D) Violin plots showing the expression of differential genes (Down‐regulated in *Pkp2*‐KO) in cardiomyocytes. All data are presented as mean ± SEM. *p < 0.05, **p < 0.01, and ***p < 0.001.
**FIGURE S5**: Expression of CF‐Related Genes and Marker Genes for CF Cluster Identification in scRNA‐seq Data. (A) Expression of select CF‐related genes as visualized on UMAP plots. (B) Dot plot visualization of top marker genes used to identify CF clusters. The color and size of the dots indicate the relative average expression level in each population and the percentage of cells expressing the gene, respectively.
**FIGURE S6**: Single‐cell sequencing analysis reveals molecular signatures and functional networks in *Pkp2*‐KO cardiomyopathy. (A) Classification of CF clusters. Representative Gene Ontology (GO) terms and CF‐related genes in each cluster were displayed. (B) Trace diagram of CF clusters. (C) Heatmap of the difference in quantity and intensity between the two samples. *Pkp2*‐KO, compared with AAV9‐PKP2 or WT, is up‐regulated in red, and *Pkp2*‐KO is down‐regulated in blue. (D) Strength difference network diagram between the two samples. *Pkp2*‐KO, compared with AAV9‐PKP2 or WT, is up‐regulated in red, and *Pkp2*‐KO is down‐regulated in blue.
**TABLE S1**: Primer sequences of related genes.

## Data Availability

All rat single‐cell RNA sequencing data (including raw FASTQ files) have been deposited in the public NCBI Sequence Read Archive (SRA) under the accession number PRJNA1290835. Similarly, all mouse transcriptome sequencing data (including raw FASTQ files) have been deposited in the public NCBI Sequence Read Archive under accession number PRJNA1290839. These datasets will become publicly accessible after August 2028.
