# Supplementary material for: Gene Therapy Targeting Pkp2 Deficiency Attenuates Cardiac Fibrosis: Insights From Single‐Cell Transcriptomics in Pkp2‐Knockout Rats
Source: MedComm (2020). 2025 Sep 18;6(10):e70392. doi: 10.1002/mco2.70392 (PMC12446705; doi:10.1002/mco2.70392)
Supplement: Supplementary file 1 — FIGURE S1: Construction of cardiac organoid model. (A) Morphological images of cardiac organoids before and after hypoxia. (B) Cardiac signature maker (cTnT, CD31, αSMA, MYH7 and α‐actinin) immunofluorescence staining image of cardiac organoid. (C) The expression of CD45 and APOE proteins in hypoxia‐damaged cardiac organoids (n = 3). All data are presented as mean ± SEM. *p < 0.05, **p < 0.01, and ***p < 0.001. FIGURE S2: Safety evaluation and metabolic level of AAV9‐PKP2 in vivo. (A) HE pathological staining of lung, liver, and kidney tissues of different groups of rats (n = 3). (B) Results of serum safety indexes (CRP, CR, ALT) in different groups (n = 5:7:7). (C) HE pathological staining of lung, liver, and kidney tissues of different groups of mice (n = 3). All data are presented as mean ± SEM. *p < 0.05, **p < 0.01, and ***p < 0.001. FIGURE S3: Long‐term expression study of AAV9 vector in the heart. (A) Cardiac immunofluorescence staining after AAV9‐zsgreen injection in rats at different times (n = 3). FIGURE S4: Characterization of cell cluster contributions and differential expression in scRNA‐seq Analysis. (A) Bar plot of the percentage of cluster contributions in each scRNA‐seq sample. (B) Dot plot visualization of top marker genes used to identify clusters. The color and size of the dots indicate the relative average expression level in each population and the percentage of cells expressing the gene, respectively. (C) Fraction of cell types. (D) Violin plots showing the expression of differential genes (Down‐regulated in Pkp2‐KO) in cardiomyocytes. All data are presented as mean ± SEM. *p < 0.05, **p < 0.01, and ***p < 0.001. FIGURE S5: Expression of CF‐Related Genes and Marker Genes for CF Cluster Identification in scRNA‐seq Data. (A) Expression of select CF‐related genes as visualized on UMAP plots. (B) Dot plot visualization of top marker genes used to identify CF clusters. The color and size of the dots indicate the relative average expression level in [file MCO2-6-e70392-s001.docx]

Gene Therapy Targeting *Pkp2* Deficiency Attenuates Cardiac Fibrosis: Insights from Single-Cell Transcriptomics in *Pkp2*-Knockout Rats

Xinyue Ding^1,#^, Hui Zhang^1,#^, Xuan Zhao^1,#^, Nengpin Yin^1^, Shuo Han^1^, Xiao Jin^1^, Tingting Li^1^, Lina Xing^3^, Zhen Qi^4^, Yanan Zhu^1^, Xin Wang^2,*^, Zongjun Liu^1,*^

*^1.^ Institute of Cardiovascular Translational Medicine, Putuo Hospital, Shanghai University of Traditional Chinese Medicine, Putuo District, Shanghai, 200062, China;*

*^2.^ Shanghai Key Laboratory of Regulatory Biology, School of Life Sciences, East China Normal University, Shanghai, 200241, China.*

*^3.^ Department of GCP Office, Shanghai Ninth People’s Hospital, Shanghai Jiao Tong University School of Medicine, Shanghai, 200011, China*

*^4.^ Neocellmed Co., Ltd., Shanghai, 201315, China.*

E-mail address:

[xwang@bio.ecnu.edu.cn](mailto:xwang@bio.ecnu.edu.cn) (X. Wang),

[liuzongjun1548@shutcm.edu.cn](mailto:liuzongjun1548@shutcm.edu.cn) (Z. Liu).

^#^These authors contributed equally to this work

**
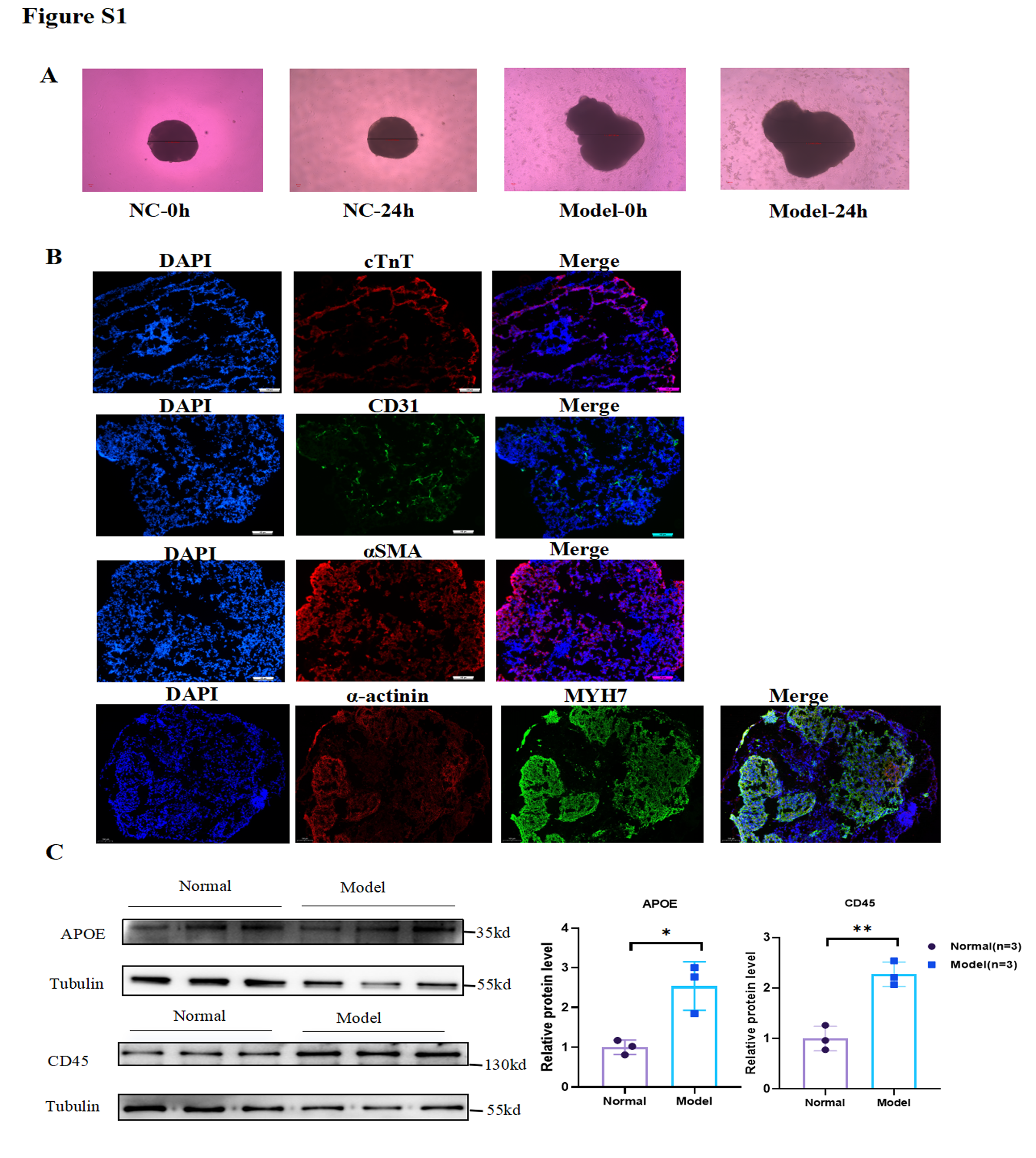
**

**Figure S1.** Construction of cardiac organoid model. (A) Morphological images of cardiac organoids before and after hypoxia. (B) Cardiac signature maker (cTnT, CD31, αSMA,MYH7 and α-actinin) immunofluorescence staining image of cardiac organoid. (C) The expression of CD45 and APOE proteins in hypoxia-damaged cardiac organoids (n=3). All data are presented as mean ± SEM. **P* < 0.05, ***P* < 0.01, and ****P* < 0.001.


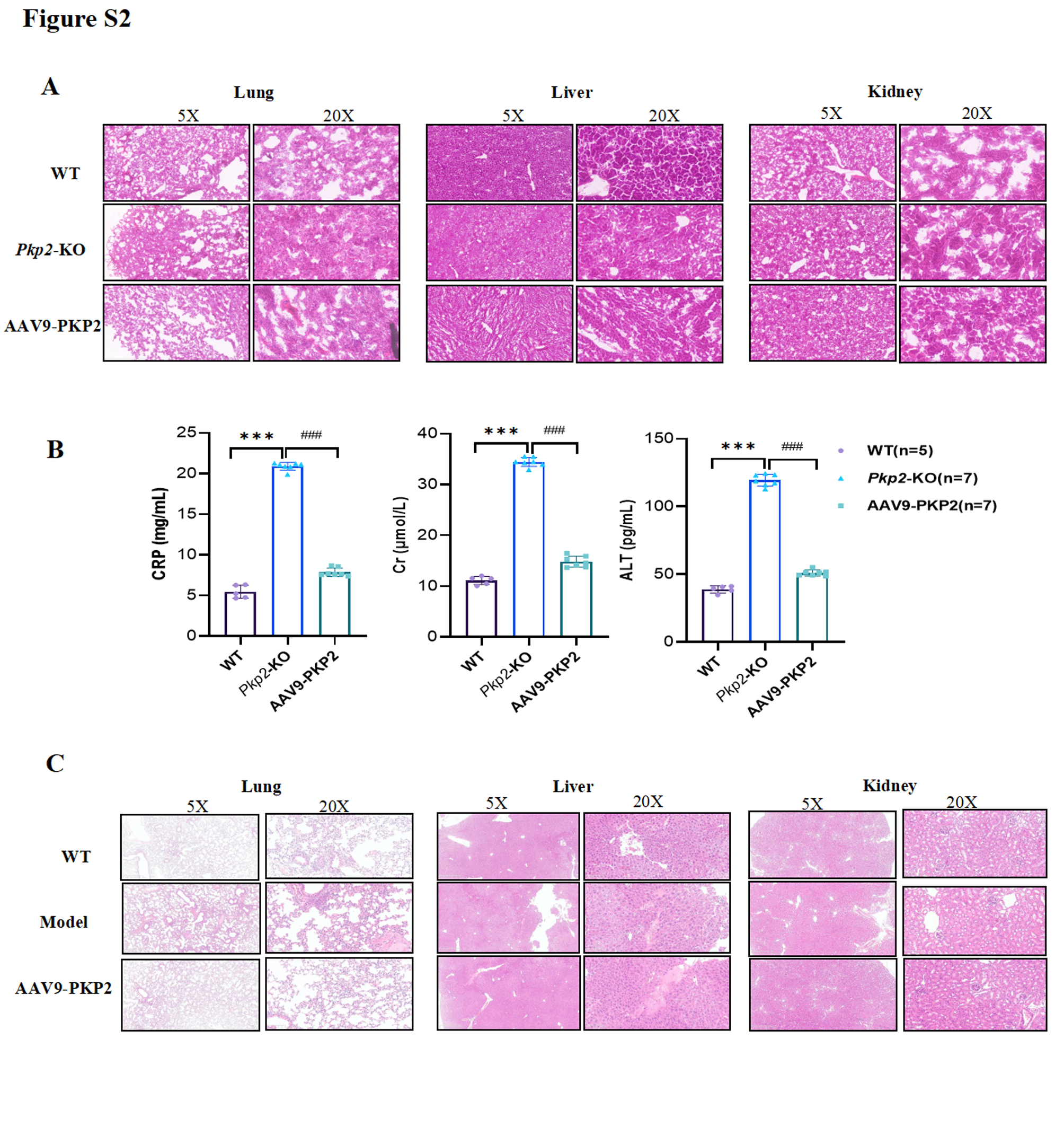


**Figure S2.** Safety evaluation and metabolic level of AAV9-PKP2 in vivo. (A) HE pathological staining of lung, liver, and kidney tissues of different groups of rats (n = 3). (B) Results of serum safety indexes (CRP, CR, ALT) in different groups (n=5:7:7). (C) HE pathological staining of lung, liver, and kidney tissues of different groups of mice (n=3). All data are presented as mean ± SEM. **P* < 0.05, ***P* < 0.01, and ****P* < 0.001.

**
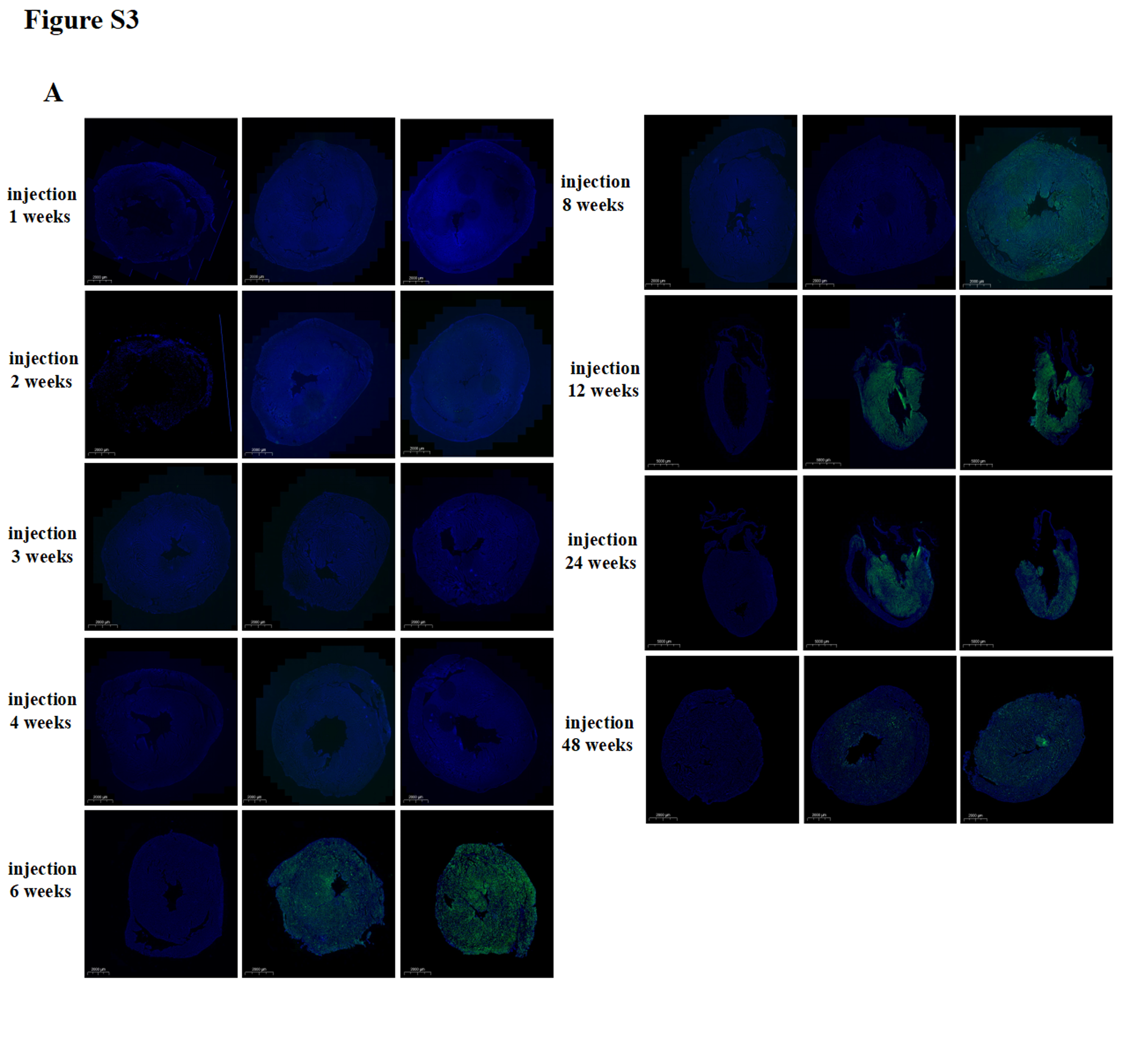
**

**Figure S3.** Long-term expression study of AAV9 vector in the heart. (A) Cardiac immunofluorescence staining after AAV9-zsgreen injection in rats at different times (n=3).


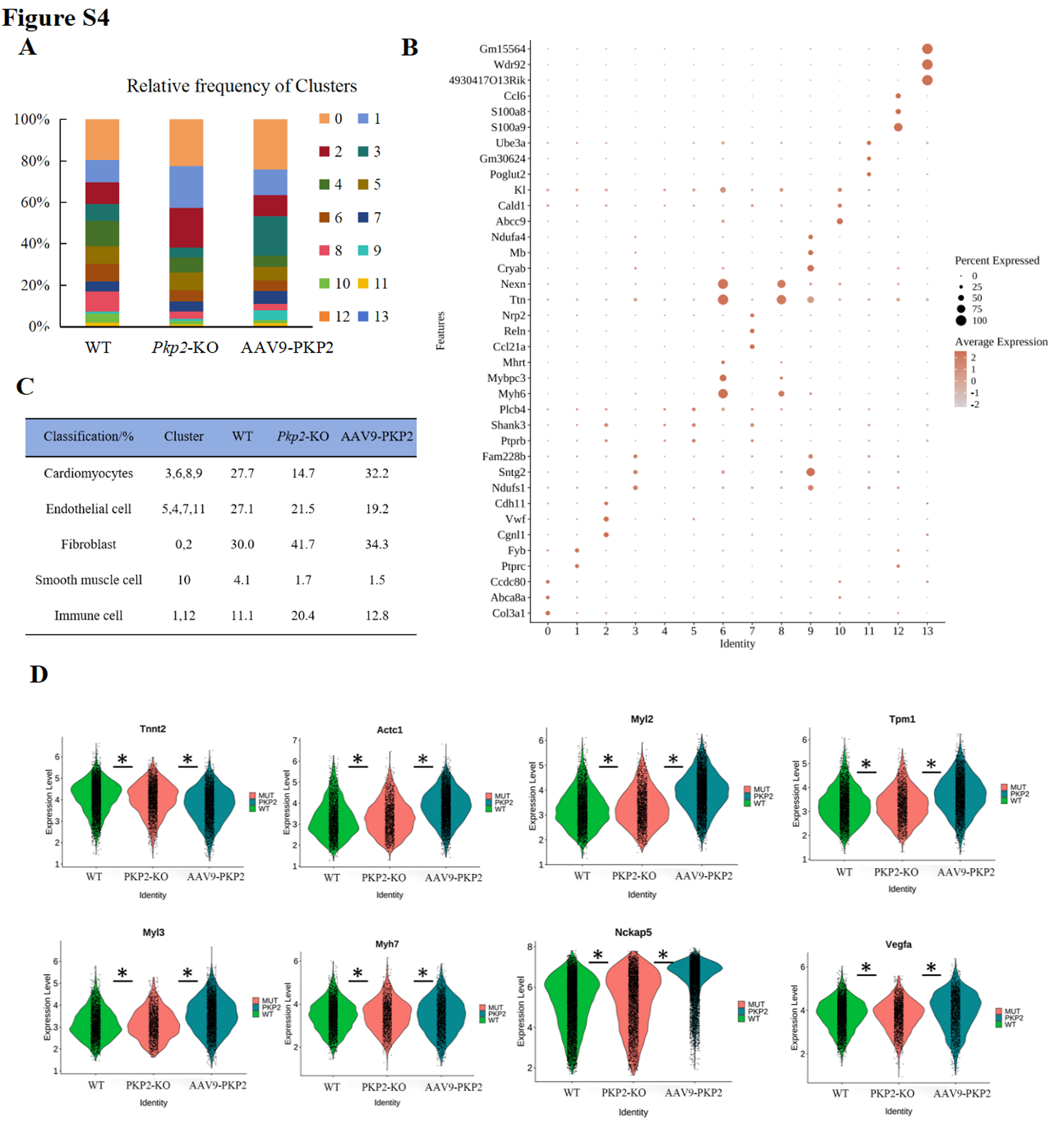


**Figure S4.** Characterization of cell cluster contributions and differential expression in scRNA-seq Analysis. (A) Bar plot of the percentage of cluster contributions in each scRNA-seq sample. (B) Dot plot visualization of top marker genes used to identify clusters. The color and size of the dots indicate the relative average expression level in each population and the percentage of cells expressing the gene, respectively. (C) Fraction of cell types. (D) Violin plots showing the expression of differential genes (Down-regulated in *Pkp2*-KO) in cardiomyocytes.All data are presented as mean ± SEM. **P* < 0.05, ***P* < 0.01, and ****P* < 0.001.


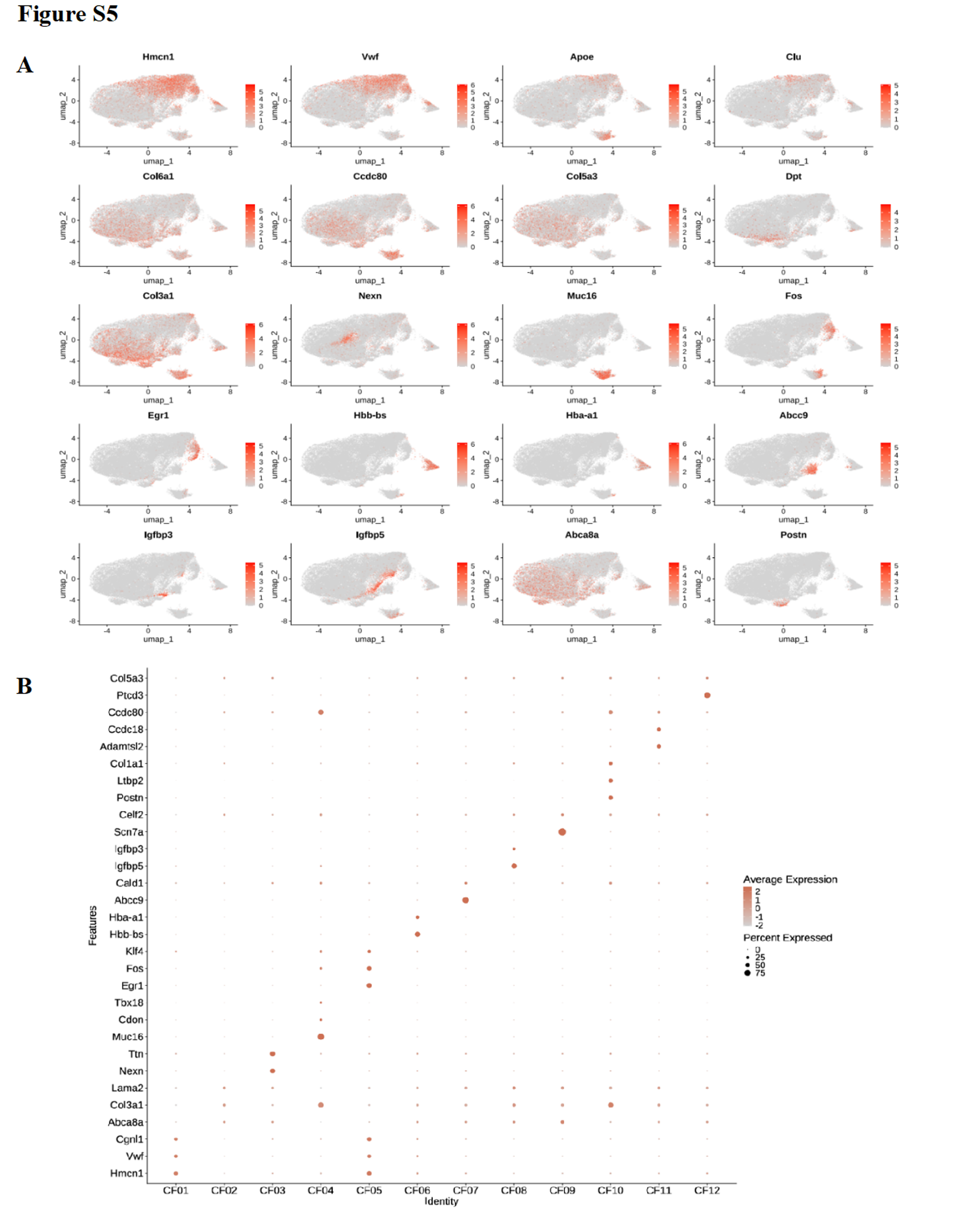


**Figure S5.** Expression of CF-Related Genes and Marker Genes for CF Cluster Identification in scRNA-seq Data. (A) Expression of select CF-related genes as visualized on UMAP plots. (B) Dot plot visualization of top marker genes used to identify CF clusters. The color and size of the dots indicate the relative average expression level in each population and the percentage of cells expressing the gene, respectively.


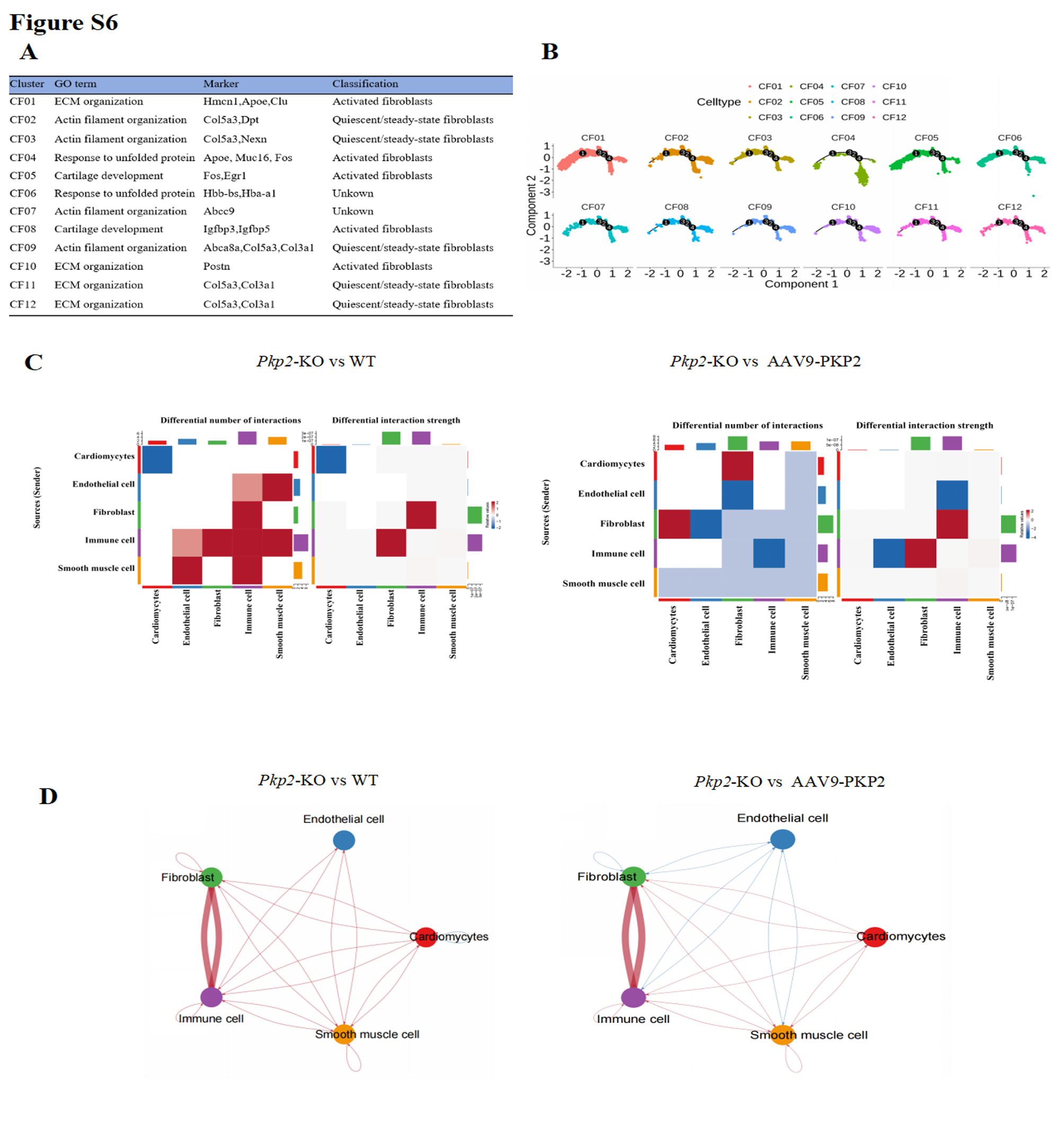


**Figure S6.** Single cell sequencing analysis reveals molecular signatures and functional networks in *Pkp2*-KO cardiomyopathy. (A) Classification of CF clusters. Representative Gene Ontology (GO) terms and CF-related genes in each cluster were displayed. (B) Trace diagram of CF clusters.(C) Heatmap of the difference in quantity and intensity between the two samples. *Pkp2*-KO, compared with AAV9-PKP2 or WT, is up-regulated in red, and *Pkp2*-KO is down-regulated in blue. (D) Strength difference network diagram between the two samples. *Pkp2*-KO, compared with AAV9-PKP2 or WT, is up-regulated in red, and *Pkp2*-KO is down-regulated in blue.


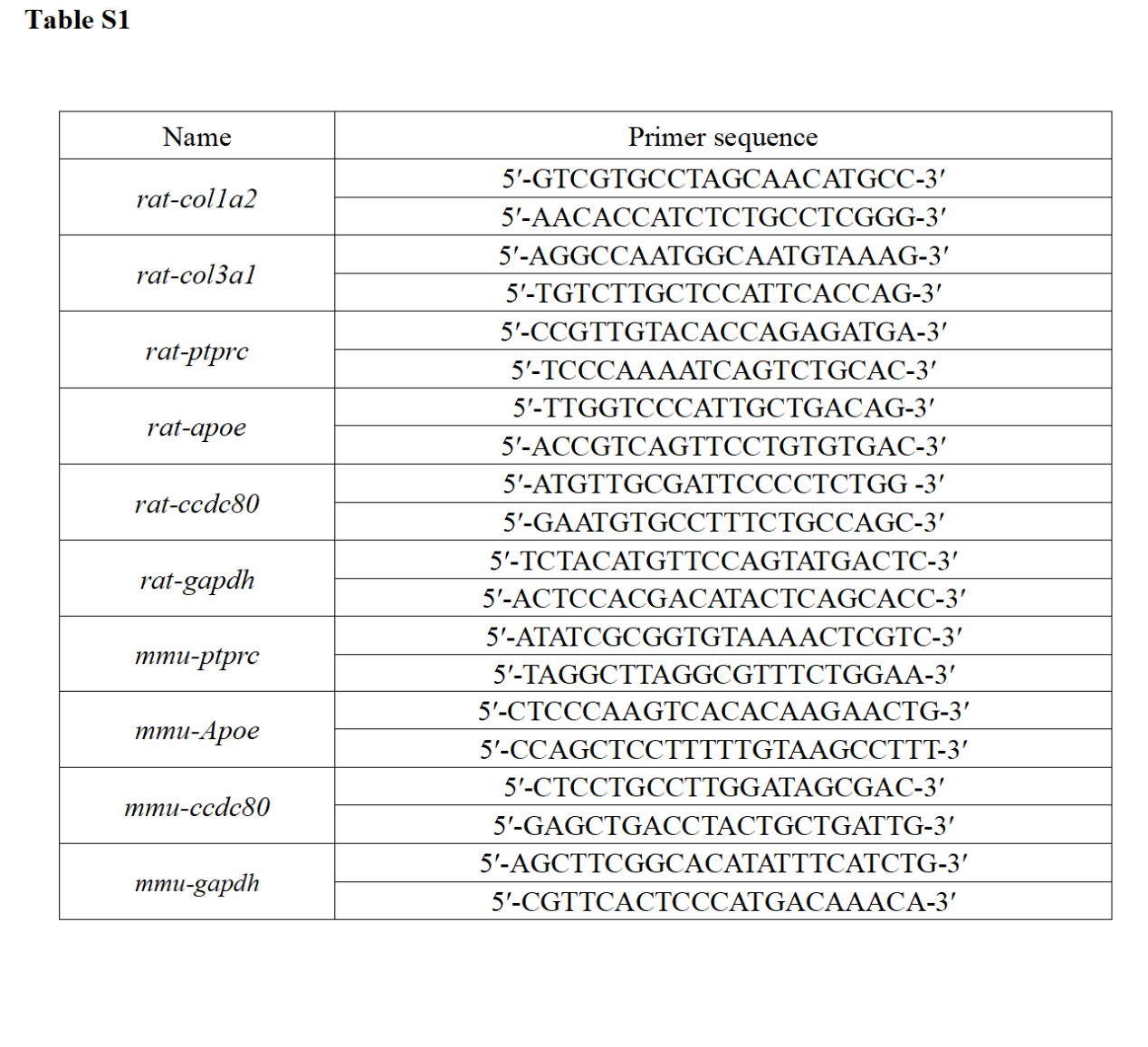


**Table S1.** Primer sequences of related genes.

**Table S1.** Primer sequences of related genes.

| Name | Primer sequence |
| --- | --- |
| *rat-col1a2* | 5′-GTCGTGCCTAGCAACATGCC-3′ |
|  | 5′-AACACCATCTCTGCCTCGGG-3′ |
| *rat-col3a1* | 5′-AGGCCAATGGCAATGTAAAG-3′ |
|  | 5′-TGTCTTGCTCCATTCACCAG-3′ |
| *rat-ptprc* | 5′-CCGTTGTACACCAGAGATGA-3′ |
|  | 5′-TCCCAAAATCAGTCTGCAC-3′ |
| *rat-apoe* | 5′-TTGGTCCCATTGCTGACAG-3′ |
|  | 5′-ACCGTCAGTTCCTGTGTGAC-3′ |
| *rat-ccdc80* | 5′-ATGTTGCGATTCCCCTCTGG -3′ |
|  | 5′-GAATGTGCCTTTCTGCCAGC-3′ |
| *rat-gapdh* | 5′-TCTACATGTTCCAGTATGACTC-3′ |
|  | 5′-ACTCCACGACATACTCAGCACC-3′ |
| *mmu-ptprc* | 5′-ATATCGCGGTGTAAAACTCGTC-3′ |
|  | 5′-TAGGCTTAGGCGTTTCTGGAA-3′ |
| *mmu-Apoe* | 5′-CTCCCAAGTCACACAAGAACTG-3′ |
|  | 5′-CCAGCTCCTTTTTGTAAGCCTTT-3′ |
| *mmu-ccdc80* | 5′-CTCCTGCCTTGGATAGCGAC-3′ |
|  | 5′-GAGCTGACCTACTGCTGATTG-3′ |
| *mmu-gapdh* | 5′-AGCTTCGGCACATATTTCATCTG-3′ |
|  | 5′-CGTTCACTCCCATGACAAACA-3′ |
